# The roles and prognostic significance of ABI1-TSV-11 expression in patients with left-sided colorectal cancer

**DOI:** 10.1038/s41598-021-90220-8

**Published:** 2021-05-24

**Authors:** Yu Zhang, Zhaohui Zhong, Mei Li, Jingyi Chen, Tingru Lin, Jie Sun, Di Wang, Qing Mu, Huiting Su, Na Wu, Aiyu Liu, Yimeng Yu, Menglei Zhang, Yulan Liu, Jingzhu Guo, Weidong Yu

**Affiliations:** 1 Department of Central Laboratory and Institute of Clinical Molecular Biology, Peking University People’s Hospital, Beijing, China; 2 Department of Gastroenterology, Peking University People’s Hospital, Beijing, China; 3 Department of General Surgery, Peking University People’s Hospital, Beijing, China; 4 Department of Animal Laboratory, Peking University People’s Hospital, Beijing, China; 5 Department of Pediatric, Peking University People’s Hospital, Beijing, China

**Keywords:** Transcriptomics, Cancer

## Abstract

Abnormally expressed and/or phosphorylated Abelson interactor 1 (ABI1) participates in the metastasis and progression of colorectal cancer (CRC). *ABI1* presents as at least 12 transcript variants (TSVs) by mRNA alternative splicing, but it is unknown which of them is involved in CRC metastasis and prognosis. Here, we firstly identified ABI1-TSV-11 as a key TSV affecting the metastasis and prognosis of left-sided colorectal cancer (LsCC) and its elevated expression is related to lymph node metastasis and shorter overall survival (OS) in LsCC by analyzing data from The Cancer Genome Atlas and TSVdb. Secondly, ABI1-TSV-11 overexpression promoted LoVo and SW480 cells adhesion and migration in vitro, and accelerated LoVo and SW480 cells lung metastasis in vivo. Finally, mechanism investigations revealed that ABI1-isoform-11 interacted with epidermal growth factor receptor pathway substrate 8 (ESP8) and regulated actin dynamics to affect LoVo and SW480 cells biological behaviors. Taken together, our data demonstrated that ABI1-TSV-11 plays an oncogenic role in LsCC, it is an independent risk factor of prognosis and may be a potential molecular marker and therapeutic target in LsCC.

## Introduction

Colorectal cancer (CRC) accounts for the third morbidity and the second mortality in cancer-related diseases in the worldwide^1^. Metastasis is still the leading cause of CRC death though advances in strategies for early diagnosis and treatment^2^ and it is a dynamic biological process that includes separation from the primary site, degradation of extracellular matrix, invasion into blood and lymphatic vessels, migration and movement in vessels, and distant colonization and neovascularization^3–5^. One or more of the above processes is blocked, the process of tumor metastasis would be inhibited. Left-sided colorectal cancer (LsCC) and right-sided colorectal cancer (RsCC) have significant differences in epidemiological characteristics, molecular biological characteristics, metastatic behavior, and disease outcome^6^. For example, Chromosomal instability is more common in LsCC patients, and the prognosis of LsCC patients in TNM II stage is worse than that of RsCC^6,7^. Therefore, stratified analysis based on LsCC and RsCC subgroups may benefit to personalized treatment and mortality reduction.

Abelson interactor 1 (ABI1) is an important adaptor protein and its abnormal expression and/or phosphorylation are involved in regulating the behaviors of tumor cells, such as proliferation, adhesion, migration and invasion, and thereby affecting the metastasis and progression of various malignant tumors, including CRC^8–13^. ABI1 regulates tumor cell proliferation by binding to c-Abl^14^, v-Abl^15^, EPS8^16^ and the p85 subunits of PI3K^17^. It also regulates cell–cell adhesion, cell–extracellular matrix adhesion, cell extension, migration and invasion of tumor cells by forming multi-protein complexes with WAVE2^18–20^, PI3K^17,21^, EPS8^11,22,23^ and N-WASP^24^. *ABI1* has been reported to serve as an oncogene in CRC^8,25–27^, leukemia^9,28^, breast cancer^10,29,30^, ovarian cancer^11,31^ and hepatocellular carcinoma^12,32^. There is also evidence suggesting that it may serve as a tumor suppressor gene in prostate cancer^18,33,34^, gastric cancer^35,36^ and neuroblastoma^37^. Recent studies have shown that ABI1 is highly expressed^25,26^ and hypophosphorylated^38^ in CRC tissues. Especially, ABI1 is located at the invasive fronts of tumor tissues^25^ and the sites of degradation of extracellular matrix^8^. There are correlations of ABI1 expression with infiltration depth and degree of differentiation of CRC tumor, and CRC patients with high ABI1 expression have a lower 5-year survival rate and poorer prognosis^25^. In addition, ABI1 pY435 is necessary for CRC cells to form lamellipodium-like cellular protrusions^8,27^.

Alternative splicing (AS), one of the post-transcriptional regulatory mechanisms, is the main factor accounting for proteomic diversity^39^. And differential transcript variants (TSVs) expression of some genes is associated with the metastasis of CRC^40,41^. *ABI1* is a multi-exon gene and has at least 12 TSVs^42^. Different ABI1-TSVs often play synergistic or antagonistic roles in the same pathophysiological events^21,43^. However, the role of ABI1-TSVs in the metastasis of CRC has not yet been investigated.

The Cancer Genome Atlas (TCGA) integrates the sequencing data of 33 types of cancer and normal samples, as well as detailed follow-up information^44^ and TSVdb (http://tsvdb.com) is an open visualization database to study the differential expression of TSVs in various tumors^45^. In this study, we set out to investigate the roles of ABI1-TSVs in regulating tumor metastasis and progression. We found a key ABI1-TSV, ABI1-TSV-11, that associates with lymph node metastasis and short overall survival (OS) and thus serves as an independent risk factor for LsCC. We proved that ABI1-TSV-11 overexpression promoted the adhesion and migration of LoVo and SW480 cells and the invasion of LoVo cells in vitro, and accelerated the lung metastasis of LoVo and SW480 cells in vivo. We also demonstrated that ABI1-isoform-11 (the ABI1-TSV-11-encoded protein isoform) interacts with EPS8 to regulate the adhesion, migration, and invasion of LoVo and SW480 cells by affecting actin dynamics.

## Results

### Elevated expression of ABI1-TSV-11 correlates with lymph node metastasis and predicts poor prognosis in patients with LsCC

To determine the clinical significance and underlying role of ABI1-TSVs in CRC and its subgroups (LsCC and RsCC), we first analyzed the correlations between the clinicopathological information (sex, age, ethnicity, TNM stage, etc.) and survival time (OS; disease-free survival, DFS) by Kaplan–Meier analysis and chi-squared test to evaluate the reliability and representativeness of data. As shown in Table 1, TNM stage was closely related to survival time (OS, DFS) and the selected cases are representative of the typical clinicopathological and prognostic characteristics of CRC patients.

**Table 1:** The correlation between clinicopathological characteristics and OS/ DFS in CRC, LsCC and RsCC patients from TCGA database.

Characteristics	OS						DFS					
	CRC		LsCC		RsCC		CRC		LsCC		RsCC	
	χ2	P	χ2	P	χ2	P	χ2	P	χ2	P	χ2	P
Sex	0.904	0.342	0.083	0.774	1.476	0.224	2.545	0.111	0.918	0.338	0.507	0.477
Age	10.675	0.001	7.283	0.007	2.125	0.145	0.055	0.815	0.018	0.893	0.280	0.597
Race	0.186	0.911	0.286	0.867	0.180	0.914	0.823	0.663	0.624	0.732	0.771	0.680
Clinical stage	19.041	0.000	10.624	0.001	10.975	0.001	20.781	0.000	9.937	0.002	11.082	0.001
Infiltration depth	2.804	0.094	0.116	0.734	5.518	0.019	9.111	0.003	1.849	0.174	6.981	0.008
Lymph-node metastasis	17.505	0.000	9.258	0.010	12.840	0.002	23.180	0.000	11.658	0.003	24.627	0.000
Distant metastasis	19.612	0.000	2.032	0.154	24.049	0.000	24.286	0.000	5.938	0.015	15.097	0.000
KRAS mutation	0.732	0.392	1.001	0.317	1.582	0.208	0.963	0.326	2.253	0.133	0.039	0.844

*CRC* colorectal cancer, *DFS* disease-free survival, *LsCC* left-sided colorectal cancer, *OS* over-all survival, *RsCC* right-sided colorectal cancer.

**Figure 1:**
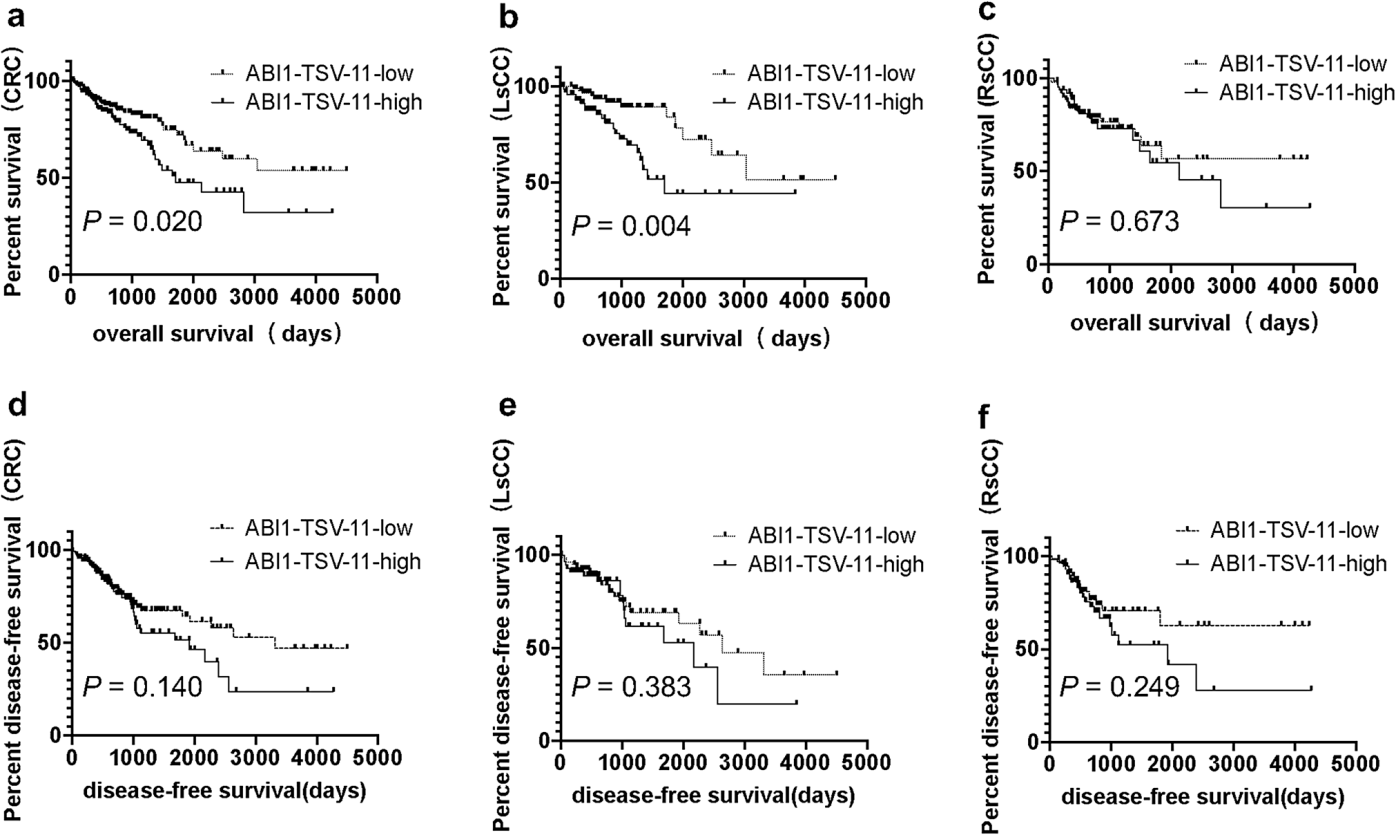
Elevated expression of ABI1-TSV-11 is related to shorter overall survival of CRC and LsCC patients. (**a**)–(**c**) Kaplan–Meier analysis of the correlation between ABI1-TSV-11 expression and overall survival in colorectal cancer (CRC, a, *n* = 339, *P* = 0.020), left-sided colorectal cancer (LsCC, b, *n* = 168, *P* = 0.004), and right-sided colorectal cancer (RsCC, c, *n* = 154, *P* = 0.673). (**d**)–(**f**) Kaplan–Meier analysis of the correlation between ABI1-TSV-11 and disease-free survival in CRC (**d**, *n* = 339, *P* = 0.140), LsCC (**e**,* n* = 168, *P* = 0.383), and RsCC (**f**, *n* = 154, *P* = 0.249); log-rank test, *P* < 0.05, significant.

Next, we found that increased ABI1-TSV-11 (Abelson interactor 1 transcript variant 11) expression correlated with lymph node metastasis (*P* = 0.050, Table 2) in LsCC, and there was no correlation between ABI1-TSV-11 overexpression and KRAS mutation. Notably, Kaplan–Meier analysis showed that, in the CRC group and LsCC group, the 10-year OS rates (32.1% and 44.3%, respectively) in patients with high ABI1-TSV-11 expression were significantly lower than in those with low ABI1-TSV-11 expression (53.8% and 51.5%, respectively; *P* = 0.020 and *P* = 0.004; Fig. 1a,b). There were no statistically significant differences in 10-year DFS rates observed between patients with high expression and low expression of ABI1-TSV-11(Fig. 1d,e). In the RsCC group, while patients with high ABI1-TSV-11 expression showed 10-year OS and DFS rates lower than those with low ABI1-TSV-11 expression, the differences were not statistically significant (Fig. 1c,f).The results of Multivariate Cox proportional hazards models showed that ABI1-TSV-11 could serve as an independent risk factor in LsCC [hazard ratio (HR) = 3.060, *P* = 0.008; Table 3], but not an independent risk factor in CRC (Table 4). Taken together, these results indicate that ABI1-TSV-11 is a specific prognostic risk factor and may serve as a therapeutic target for patients with LsCC.

**Table 2:** The correlation between ABI1-TSV-11 expression and clinicopathological characteristics of CRC, LsCC and RsCC patients from TCGA.

Characteristics	CRC				LsCC				RsCC			
	n	Low/high	χ2	P	n	Low/high	χ2	P	n	Low/high	χ2	P
**Sex**	339		3.027	0.082	168		2.914	0.088	154		1.765	0.184
Male		98/88				41/43				48/42		
Female		95/58				52/32				41/23		
**Age**	339		1.039	0.308	168		5.154	0.023	154		0.499	0.480
< 65		102/69				62/37				36/30		
≥ 65		91/77				31/38				53/35		
**Race**	309		0.328	0.849	149		2.741	0.254	143		2.673	0.263
Asian		5/5				3/0				2/5		
Black		34/23				12/7				22/14		
White		140/102				70/57				59/41		
**Clinical stage**	323		0.207	0.649	160		0.243	0.622	146		0.027	0.869
I + II		100/72				45/34				49/36		
III + IV		84/67				43/38				36/25		
**Infiltration depth**	337		0.985	0.321	167		1.374	0.241	154		0.001	0.977
T1 + T2		39/23				19/10				19/14		
T3 + T4		154/12				74/64				70/51		
**Lymph-node metastasis**	337		2.550	0.279	166		5.985	0.050	154		0.073	0.964
N0		107/78				47/36				54/39		
N1		55/35				36/21				15/12		
N2		30/32				9/17				20/14		
**Distant metastasis**	275		0.025	0.874	141		0.132	0.717	121		0.330	0.566
M0		129/99				65/51				59/44		
M1		26/21				15/10				9/9		
**KRAS mutation**	55		0.009	0.925	28			0.084^a^	21			0.080^b^
No		18/9				9/6				8/2		
Yes		19/9				12/1				4/7		

a–b fisher exact test.All others are Chi-square tests.

*CRC* colorectal cancer, *DFS* disease-free survival, *LsCC* left-sided colorectal cancer, *OS* over-all survival, *RsCC* right-sided colorectal cancer.

**Table 3:** Univariate and multivariate analysis of factors associated with overall survival in 168 LsCC patients.

Variables	Univariate analysis			Multivariate analysis		
	HR	95%CI	P	HR	95%CI	P
T (T1-T2/T3-T4)	1.184	0.448–3.130	0.734			
N (N0, N1, N2)	1.953	1.239–3.079	0.004			
M (M0, M1)	1.927	0.770–4.826	0.161			
Clinical stage (I-II/ III-IV)	3.701	1.601–8.555	0.002	6.384	2.590–15.734	0.000
ABI1-TSV-11	2.787	1.351–5.751	0.006	3.060	1.346–6.955	0.008
Age	2.632	1.268–5.465	0.009	4.072	1.768–9.370	0.001

**Table 4:** Univariate and multivariate analysis of factors associated with survival in 339 total CRC patients.

Variables	Univariate analysis			Multivariate analysis		
	HR (Hazard Ratio)	95%CI (Confidence interval)	P	HR	95%CI	P
T (T1-T2/T3-T4)	1.854	0.890–3.863	0.099			
N (N0, N1, N2)	1.825	1.399–2.381	0.000			
M (M0, M1)	3.230	1.866–5.591	0.000			
Clinical stage (I-II/III-IV)	2.870	1.750–4.708	0.000	2.687	1.608–4.490	0.000
ABI1-TSV-11	1.690	1.082–2.640	0.021			
Age	2.187	1.351–3.539	0.001	3.248	1.973–5.347	0.000

### The construction of stable cell lines overexpressing ABI1-TSV-11

**Figure 2:**
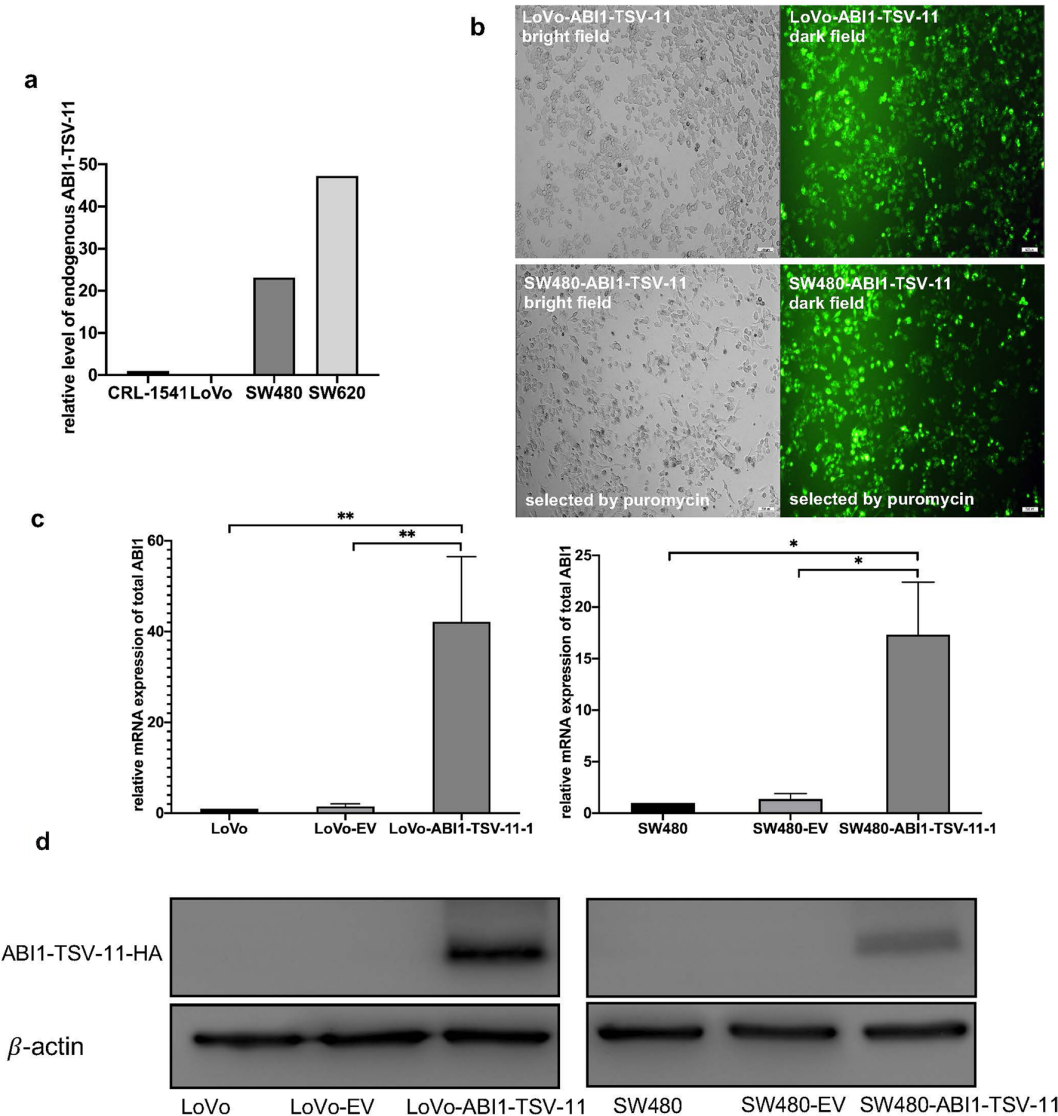
The construction of ABI1-TSV-11-overexpressing LoVo and SW480 cell models in vitro. (**a**) Relative expression levels of endogenous ABI1-TSV-11 in the normal colorectal cell line (CRL-1541) and CRC cell lines (LoVo, SW480, SW620) (RNA sequencing). (**b**) and (**c**) fluorescence images and quantitative real-time PCR analysis using *ABI1* universal primers of in LoVo-ABI1-TSV-11 and SW480-ABI1-TSV-11 cell lines after lentivirus infection and puromycin selection, as well as their controls. (**d**) Western blotting analysis of ABI1-TSV-11 in LoVo-ABI1-TSV-11 and SW480-ABI1-TSV-11 cell lines and their corresponding controls using HA antibody.

To determine the biological function of ABI1-TSV-11 in LsCC, we first analyzed the expression of ABI1-TSV-11 in a normal colorectal epithelial cell line (CRL-1541) and three CRC cell lines (LoVo, SW480, SW620) by next-generation sequencing (Fig. 2a), and LoVo and SW480 cell lines, which originate from LsCC and have low endogenous ABI1-TSV-11 expression and different invasiveness, were then selected, to construct the stable ABI1-TSV-11 overexpressed cell models. As shown in Fig. 2b–d, quantitative real-time PCR (qRT-PCR) and western blotting confirmed the successful construction of stable cell lines at the mRNA and protein levels. As shown in Fig. S1, EPS8 Knockdown were confirmed at the mRNA by qRT-PCR.

### ABI1-TSV-11 overexpression promotes LoVo and SW480 adhesion, migration, and invasion in vitro

**Figure 3:**
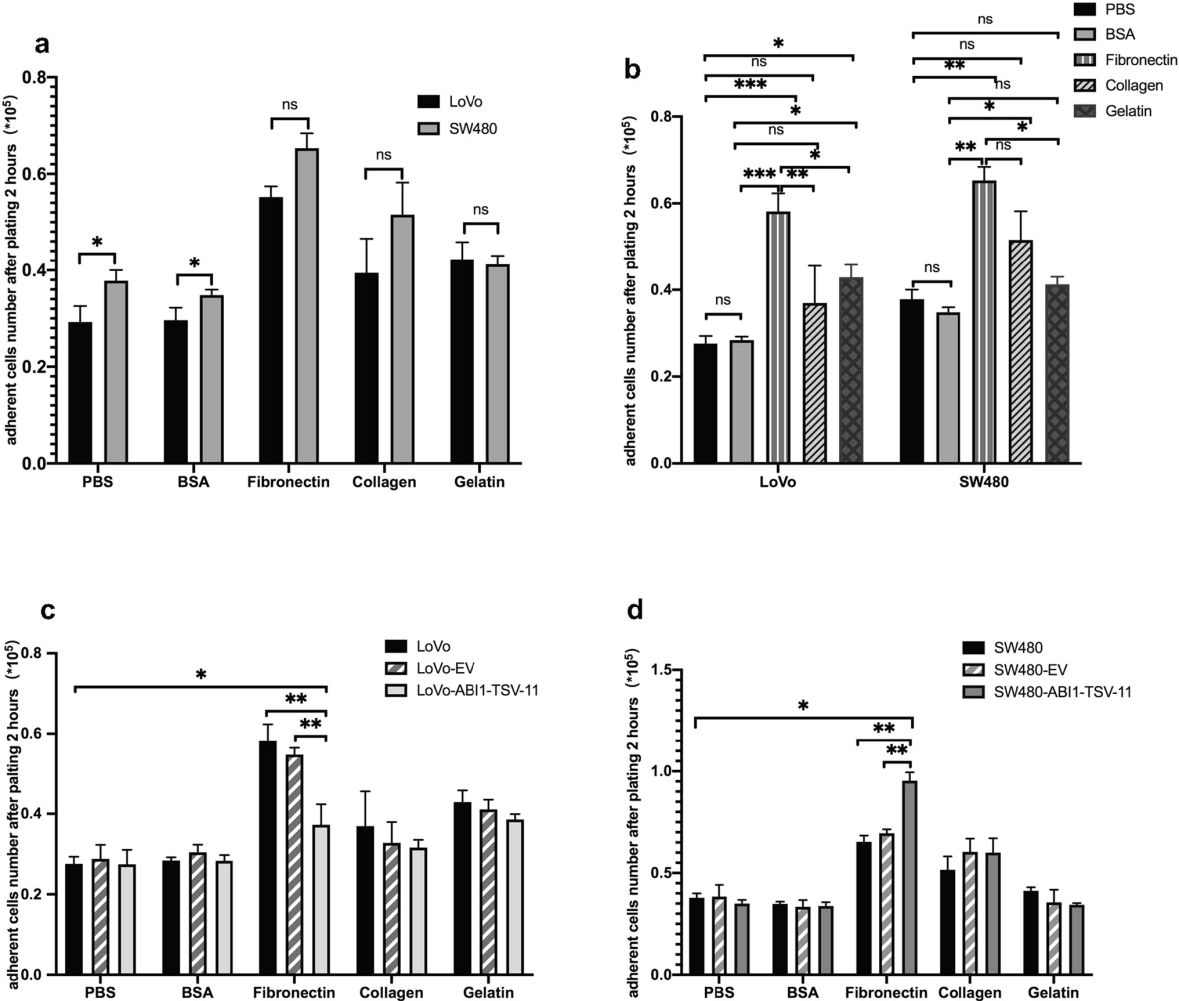
ABI1-TSV-11 overexpression enhances the cell–fibronectin adhesion of LoVo and SW480 cells in vitro. (**a**) Comparison of the adhesion ability between LoVo and SW480 cells under different coating conditions. (**b**) Changes in the adhesion ability of LoVo and SW480 cells under different coating conditions. (**c**) ABI1-TSV-11 overexpression enhanced the cell–fibronectin adhesion of LoVo cells. (**d**) ABI1-TSV-11 overexpression enhanced the cell–fibronectin adhesion of SW480 cells (one-way ANOVA, *P < 0.05, **P < 0.01, ***P < 0.001, *ns*, not significant).

To investigate the effects of ABI1-TSV-11 overexpression on cell adhesion and motility, we performed adhesion, Transwell migration, and invasion assays in vitro. For the adhesion, we firstly compared the adhesion ability of LoVo and SW480 cells under routine culture conditions (Fig. 3a) and found that the adhesion ability of LoVo cells was significantly weaker than that of SW480 cells on PBS- or 0.02% BSA-coated plates (*P* < 0.05). Next, we compared the adhesion ability of the two types of cell on fibronectin-, gelatin-, and collagen I-coated culture plates, and found that although their adhesion ability was significantly enhanced compared with that of PBS and 0.02% BSA (*P* < 0.05), their responses to stimulation with fibronectin, gelatin, and collagen I were not significantly different (Fig. 3a,b).

As shown in Fig. 3c,d, ABI1-TSV-11 overexpression did not affect the adhesion of LoVo and SW480 cells on PBS-, 0.02% BSA-, collagen I-, and gelatin-coated plates. However, ABI1-TSV-11 overexpression significantly enhanced the adhesion of LoVo and SW480 cells on fibronectin-coated plates (Fig. 3c). On the fibronectin-coated culture plates, the LoVo and SW480 cells overexpressing ABI1-TSV-11 showed different responses to fibronectin. While the overexpression of ABI1-TSV-11 inhibited LoVo cell adhesion to fibronectin, it promoted that of SW480 cells (Fig. 3c,d). To address why ABI1-TSV-11 over-expression in the LoVo and SW480 cells induces distinct responses on adhesion to fibronectin, we analyzed the mRNA expression profile of integrins using RNA-seq data of LoVo and SW480 cells, and found that the differential expression of these integrins, especially ITGB1, in SW480 and LoVo cells induces distinct responses on adhesion to fibronectin (Fig. S2).

**Figure 4:**
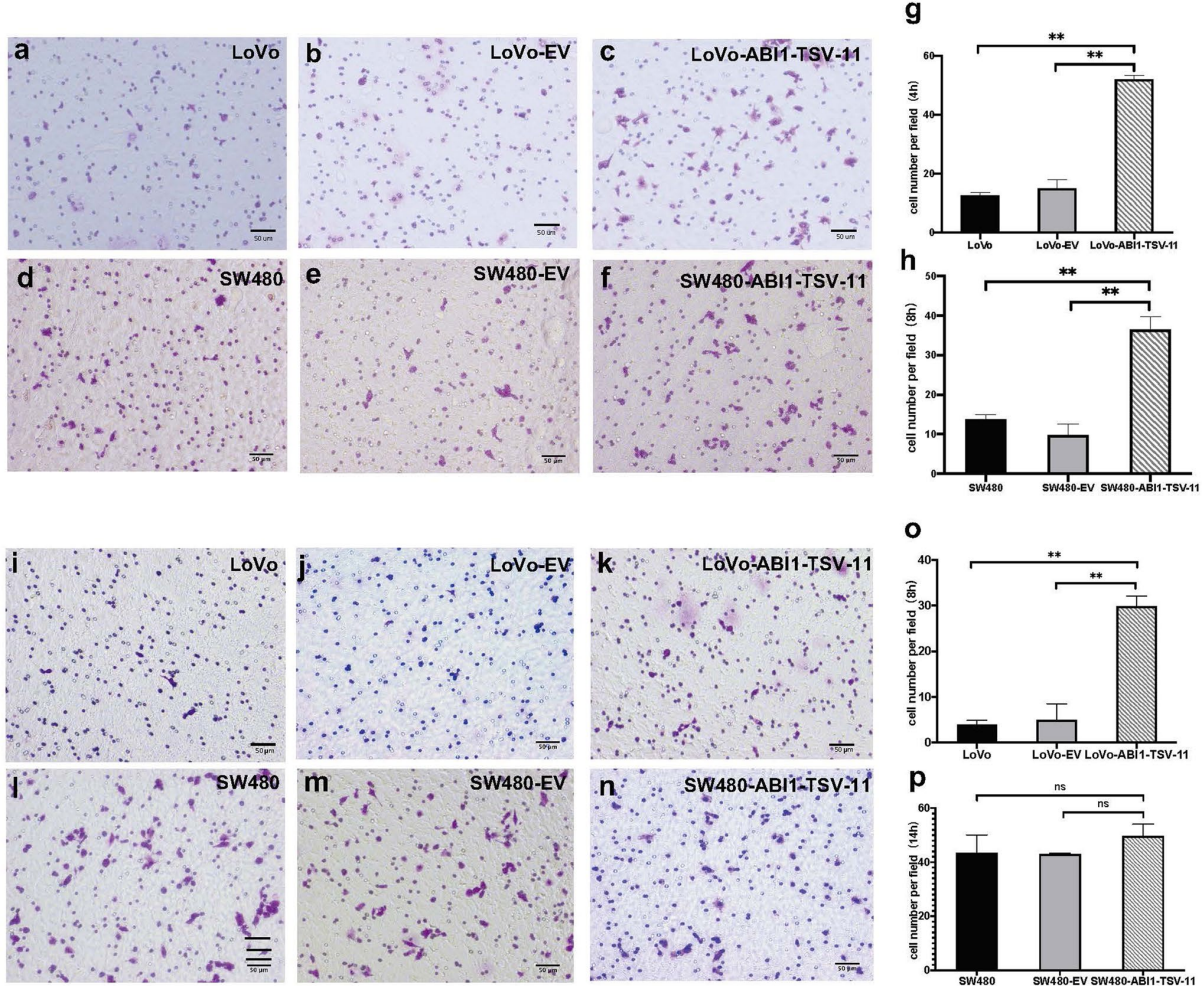
ABI1-TSV-11 overexpression enhances LoVo and SW480 cell migration and LoVo cell invasion in vitro. (**a**)–(**c**) Microscopic fields of LoVo-ABI1-TSV-11 cells and their control cells after migrating for 4 h. (**d**)–(**f**) Microscopic fields of SW480-ABI1-TSV-11 cells and their control cells after migrating for 8 h. (**j**)–(**k**) Microscopic fields of LoVo-ABI1-TSV-11 cells and their control cells after invading for 8 h. (**l**)–(**n**) Microscopic fields of SW480-ABI1-TSV-11 cells and their control cells after invading for 14 h. (**g**)–(**h**) Quantitative analysis of the effect of ABI1-TSV-11 overexpression on LoVo and SW480 cells’ migratory ability. (**o**)–(**p**) Quantitative analysis of the effect of ABI1-TSV-11 on LoVo and SW480 cells’ invasive ability (one-way ANOVA, *P < 0.05, **P < 0.01, ***P < 0.001, *ns*, not significant).

For migration and invasion, we found that ABI1-TSV-11 overexpression increased the migration and invasion of LoVo-ABI1-TSV-11 by 4.57- and 5.96-fold, respectively, compared with those of their parental control cells (both *P* < 0.05; Fig. 4g,o). ABI1-TSV-11 overexpression increased the migration of SW480-ABI1-TSV-11 cells by 17% (Fig. 4h, *P* < 0.05), but was not sufficient to stimulate the invasion of SW480-ABI1-TSV-11 cells (Fig. 4p, *P* > 0.05). To address why ABI1-TSV-11 over-expression in the LoVo and SW480 cells induces distinct responses on Matrigel invasion, we analysis the mRNA expression of MMP2 and MMP9, and found that ABI1-TSV-11 overexpression in LoVo cells could significantly increase the mRNA expression of MMP2 and MMP9, but not change the mRNA expression of them in SW480 cells. That means that it is these differences induces distinct responses on Matrigel invasion (Fig. S3).

### ABI1-TSV-11 overexpression has no influence on the proliferation of LoVo and SW480 cell lines in vitro

To test the role of ABI1-TSV-11 in LoVo and SW480 proliferation, we conducted Cell Counting Kit-8 viability assays. As shown in Fig. S4a and S4b, the viability of LoVo-ABI1-TSV-11 and SW480-ABI1-TSV-11 cell lines did not differ significantly at each time point (0, 24, and 48 h) compared with that of their corresponding controls (*P* > 0.05 at each time point). Therefore, ABI1-TSV-11 overexpression had no influence on the proliferation of LoVo and SW480 cell lines.

### ABI1-TSV-11 enhances lung metastasis of LoVo and SW480 cells in vivo

**Figure 5:**
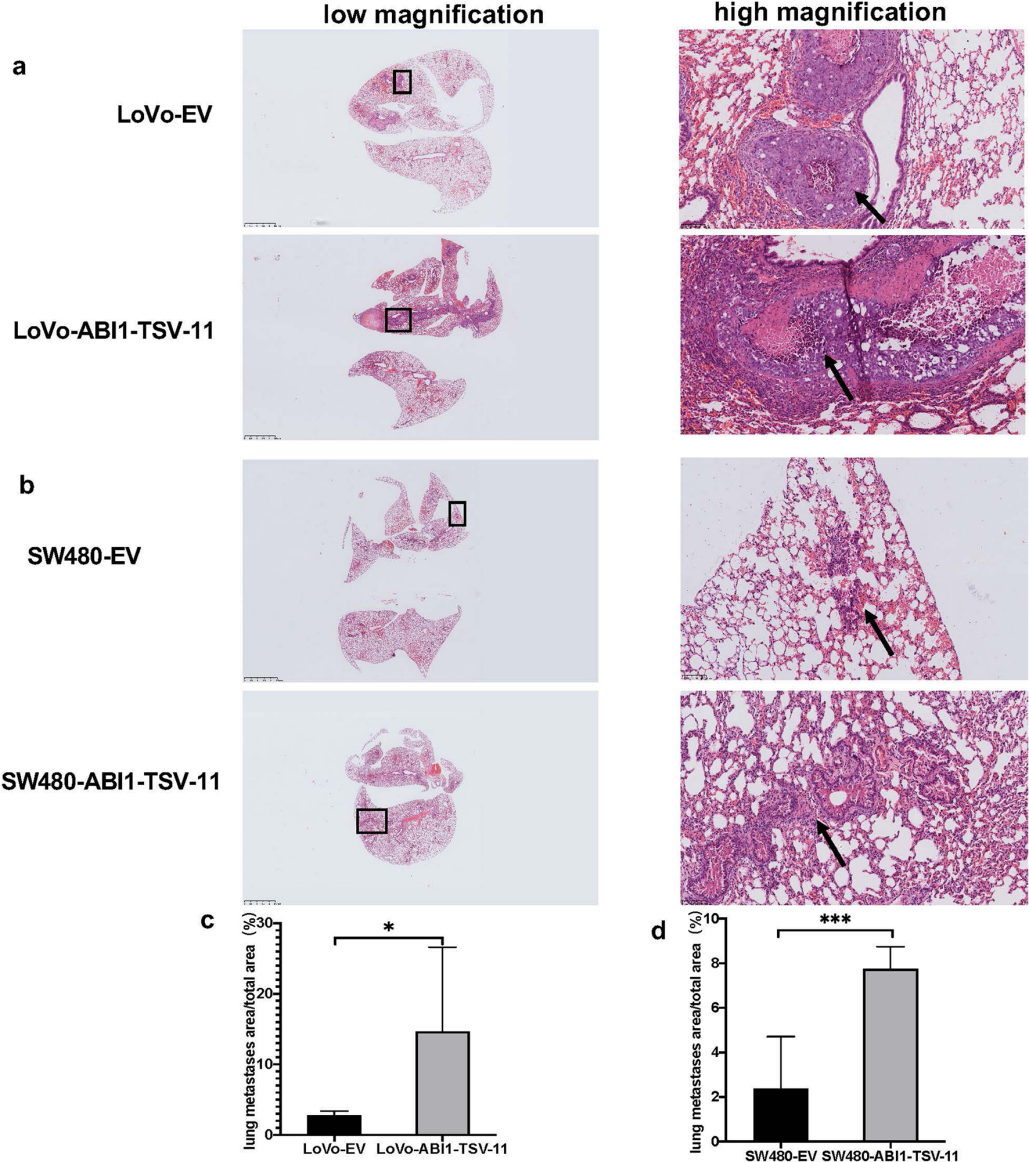
ABI1-TSV-11 overexpression promotes lung metastasis of LoVo and SW480 cells in vivo*.* (**a**) Lung HE staining images of LoVo-EV and LoVo-ABI1-TSV-11 cells. (**b**) Lung HE staining images of SW480-EV and SW480-ABI1-TSV-11 cells. (**c**,**d**) Quantitative analysis of lung metastatic area between EV groups and ABI1-TSV-11 overexpression groups. Arrows indicate the metastatic foci (*t-test.* *P < 0.05, **P < 0.01, ***P < 0.001, *ns*, not significant).

To further verify that ABI1-TSV-11 promotes LoVo and SW480 cell migration and invasion in vivo, we established lung metastasis models by intravenously injecting LoVo-ABI1-TSV-11, LoVo-EV, SW480-ABI1-TSV-11, and SW480-EV cells into the caudal veins of immunodeficient nude mice. As shown in Fig. 5a,b, the lung metastatic lesions of mice injected with LoVo-ABI1-TSV-11 and SW480-ABI1-TSV-11 cells were more severe than those injected with LoVo-EV and SW480-EV cells, respectively. In addition, the quantitative analysis showed that average proportions of lung metastatic area of the LoVo-ABI1-TSV-11 group and SW480-ABI1-TSV-11 group were 14.7% and 7.7%, while those in the LoVo-EV group and SW480-EV group were 2.8% and 2.4%. There were significant differences in this regard between LoVo-ABI1-TSV-11 and LoVo-EV, and between SW480-ABI1-TSV-11 and SW480-EV (both *P* < 0.05, Fig. 5c,d).

### ABI1-isoform-11 affects actin dynamics of LoVo and SW480 cells by interacting with EPS8

As shown in Fig. S5 and S5b, to determine how ABI1-TSV-11 promotes LsCC metastasis, we firstly proved its overexpression did not alter WAVE2, PI3K, EPS8, and N-WASP proteins levels, and ABI1-isoform-11 could only interact with EPS8, but not with WAVE2, PI3K and N-WASP in both LoVo-ABI1-TSV-11 and SW480-ABI1-TSV-11 cells. Moreover, cellular immunofluorescence analysis proved that ABI1-isoform-11 colocalized with EPS8 at the subcellular level in LoVo-ABI1-TSV-11 and SW480-ABI1-TSV-11 cells (Fig. 7a,b), which was direct evidence of the interaction between them. Meanwhile, we also observed the co-localization of ABI1-isoform-11 and F-actin (Fig. 7a,b). Taken together, the above results indicate that the interaction of ABI1-isoform-11, ESP8, and F-actin may be the mechanism by which ABI1-TSV-11 overexpression promoted the migration and invasion of LoVo-ABI1-TSV-11 and SW480-ABI1-TSV-11 cells.

To illustrate the relationship among ABI1-isoform-11, ESP8, and actin dynamics, we examined cell morphology, protrusions, and the co-localization of ABI1-isoform-11, EPS8, and F-actin when stimulated with PBS, fibronectin, and gelatin, respectively. We found that LoVo-ABI1-TSV-11 and SW480-ABI1-TSV-11 cells showed more protrusions and extensions when stimulated with fibronectin (Fig. 8b,e), while showing a round morphology and fewer protrusions when stimulated with PBS and gelatin (Fig. 8a,c,d,f). Moreover, the co-localization of ABI1-isoform-11, EPS8, and F-actin was mainly distributed on lamellipodium-like cellular protrusions and the direction of cell extension when simulated with fibronectin (Fig. 8b,e), while being mainly located at the position of cell–cell adhesion when stimulated with PBS and gelatin (Fig. 8a,c,d,f). Thus, we concluded that the interaction between ABI1-isoform-11 and EPS8 was consistent with actin dynamics in LoVo and SW480 cells. Finally, we analyzed the difference in co-localization of ABI1-isoform-11, EPS8, and F-actin in LoVo-ABI1-TSV-11 and SW480-ABI1-TSV-11 cells when stimulated with fibronectin. As shown in Fig. 8b,e, the co-localization of signals between ABI1-isoform-11 and F-actin was weaker and the co-localization between ABI1-isoform-11 and EPS8 was only in a limited punctate pattern in SW480-ABI1-TSV-11 cells compared with that in LoVo-ABI1-TSV-11 cells.

### Knockdown of EPS8 in ABI1-TSV-11-expressing LoVo or SW480 cells blocks cell migration and invasion, affect ABI1-TSV-11-induced cell morphology, protrusions, and the co-localization of ABI1-isoform-1 with F-actin

As shown in Fig. S1, EPS8 Knockdown models of LoVo and SW480 (with over-expressed ABI1-TSV-11) cells were successfully constructed and then migration and invasion assays were performed. We found the EPS8 Knockdown attenuates the migration of LoVo-ABI1-TSV-11 (Fig. S9) and SW480-ABI1-TSV-11 cells but not the invasion (Fig. S10) of them. We also found that EPS8 Knockdown did not change the mRNA expression of MMP2 and MMP9 (Fig. S11). As shown in Fig. S12, the knockdown of EPS8 in ABI1-TSV-11-expressing LoVo or SW480 cells do affect ABI1-TSV-11-induced protrusions, and the co-localization of ABI1-isoform-11 with F-actin but not cell morphology. EPS8 knockdown in ABI1-TSV-11-expressing LoVo or SW480 cells can cause ABI1-TSV-11 not to co-locate with F-actin well (arrow indicated).

## Discussion

Although clinical and experimental studies have shown that ABI1 plays an oncogenic role in CRC, high expression or low/high phosphorylation can induce the formation of lamellar pseudopodia and the degradation of extracellular matrix in CRC cells, thus promoting the clinical metastasis of CRC^8,25–27,38^, these studies have not included the roles of ABI1-TSVs.To date, research on the role of ABI1 in CRC has mainly focused on the establishment of clinical correlations and the analysis of cell phenotypes in vitro, and had no evidence of animal phenotypes *in vivo*^8,25–27,38^. Most of clinical studies used immunohistochemistry and western blotting, but the existing commercial antibodies could not specifically distinguish the isoforms encoded by the ABI1-TSVs. In vitro cytology-based studies have been unable to achieve specific analysis of one or several ABI1-TSVs, so the existing studies could not fully elucidate the molecular mechanism by which ABI1 promotes CRC metastasis, and the application of early diagnosis, prognostic evaluation, and targeted intervention for CRC based on ABI1 is also greatly limited.

RNA sequencing is the current gold standard for qualitative and quantitative analyses of transcription variants^46^. In this study, we identified for the first time that elevated expression of ABI1-TSV-11 was related to LsCC lymph node metastasis and shorter OS, and it functions as an independent risk factor of poor prognosis in LsCC. This is consistent with the conclusion in previous studies that ABI1 acts as an oncogene in CRC^8,25–27^. KRAS mutation functions as an important marker of CRC metastasis^47^. Our study found that the expression level of ABI1-TSV-11 has no correlation with KRAS mutation, which is consistent with the conclusion reached by Sebesty’en et al.^48^. It may be suggested that the effect of ABI1-TSV-11 on the metastasis of LsCC is independent of KRAS mutation. On the other hand, because the sample size available in TCGA database is relatively small (55 cases), our analysis cannot exclude the correlation between ABI1-TSV-11 and KRAS. In addition, we need to further explore the role of ABI1-TSV-11 in the cells carrying wild-type KRAS outside LoVo and SW480 cell lines^49^.

**Figure 6:**
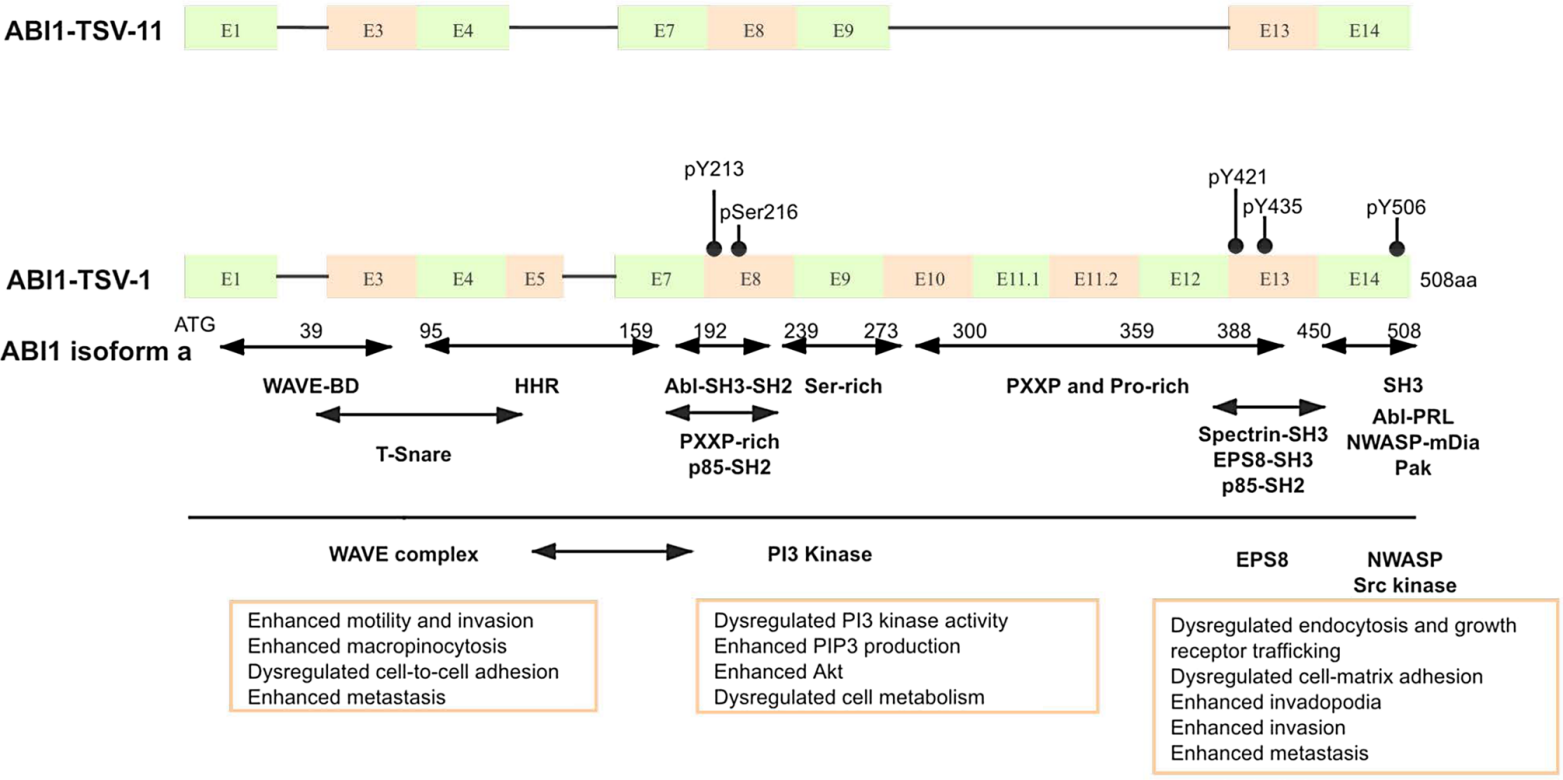
Schematic comparison of the exons and functional domains of ABI1-TSV-11 and the full-length ABI1 (ABI1-TSV-1/isoform a). (**a**) ABI1-TSV-11 has conserved WAVE2-binding and SH3 domains, but lacks the homeo-domain homologous region (HHR) and a majority of the PxxP- and Pro-rich domain. E: exon, pY213/421/435/506: phosphorylation at tyrosine 213/421/435/506, pSer216: phosphorylation at serine 216. WAVE-BD: WASP family verprolin-homologous protein-binding domain, HHR: homeodomain homologous region, Abl-SH3-SH2: Abl-Src homology 3-Src homology 2, Ser-rich: Serine-rich, SH3: Src homology 3. Modified from Dubielecka et al.^21^.

Here, we proved that ABI1-TSV-11 overexpression does not affect the proliferation ability of LoVo and SW480 cells. It has been shown that the effect of ABI1 on cell proliferation is mainly achieved through the formation of a protein complex with PI3K (p85 subunit) and activation of the PI3K pathway^17^. As Fig. 6 shows, ABI1-isoform-11 lacks HHR and PxxP proline-rich domain, it cannot structurally combine with the p85 subunit of PI3K, resulting in the failure to activate LoVo and SW480 cell-related proliferation signal pathways.

Cell adhesion mainly occurs in the forms of cell–extracellular matrix and cell–cell adhesion, and a change of cell adhesion is necessary for tumor cells to obtain the ability to metastasize^50^. Change of the adhesion to fibronectin can also directly affect the movement of various tumor cells^51,52^. Recently, Steinestel also proved that ABI1-Y435 site phosphorylation can promote the adhesion of CRC cell CDH1 to fibronectin^8^. Our study found that the overexpression of ABI1-TSV-11 can significantly increase the adhesion of SW480 cells to fibronectin and inhibit the adhesion of LoVo cells to fibronectin, but compared with PBS and 0.02% BSA, the overexpression of ABI1-TSV-11 can improve the adhesion of SW480 and LoVo cells to fibronectin. That is to say, the integrated effect of ABI1-TSV-11 overexpression is still to enhance the adhesion of SW480 and LoVo cells. It makes it easier to obtain the traction that drives cell movement, and facilitates retention and implantation in the lung.

Migration and invasion represent the key cell biological events of tumor cell metastasis^3^. The increase and/or phosphorylation of ABI1 can promote the migration and invasion of leukemia cells^9,53^, breast cancer cells^10,29^, ovarian cancer cells^11^ and liver cancer cells^12^. In CRC, the knockdown of ABI1 significantly reduced the degradation of extracellular matrix in CRC CHD1 cells, while ABI1 Y435-phosphorylation promoted the formation of lamellar pseudopodia and the invasion of extracellular matrix in CRC cells^8^. Here, we found that, although ABI1-TSV-11 can enhance the migration ability of LoVo and SW480 cells, it can only enhance the invasiveness of LoVo cells without affecting that of SW480 cells (as shown in Fig. 4). LoVo cells originated from metastasis of the upper left clavicle, with high malignancy, strong metastasis, and strong invasion^54,55^, while SW480 cells originated from a primary tumor of rectal adenocarcinoma, with low malignancy and metastatic potential^56^. The difference in the way in which ABI1-TSV-11 overexpression affected the invasiveness of LoVo and SW480 cells also confirms the results obtained in our previous clinical studies, further indicating that ABI1-TSV-11 could be an important candidate target for the treatment of LsCC metastasis.

To date, in vivo study on the role of ABI1 in tumor metastasis has been mainly related to the study of leukemia, breast cancer, and liver cancer^9,10,12,57^, but there are no related reports on CRC. Here, we chose a lung metastasis model of nude mice to evaluate the effect of ABI1-TSV-11 on the metastasis of CRC cells in vivo. The results showed that ABI1-TSV-11 overexpression promoted the process of lung metastasis of LoVo and SW480 cells in vivo, which was consistent with the results of in vitro experiments, and further proved that the overexpression of ABI1-TSV-11 enhanced the metastatic ability of LoVo and SW480 cells.

**Figure 7:**
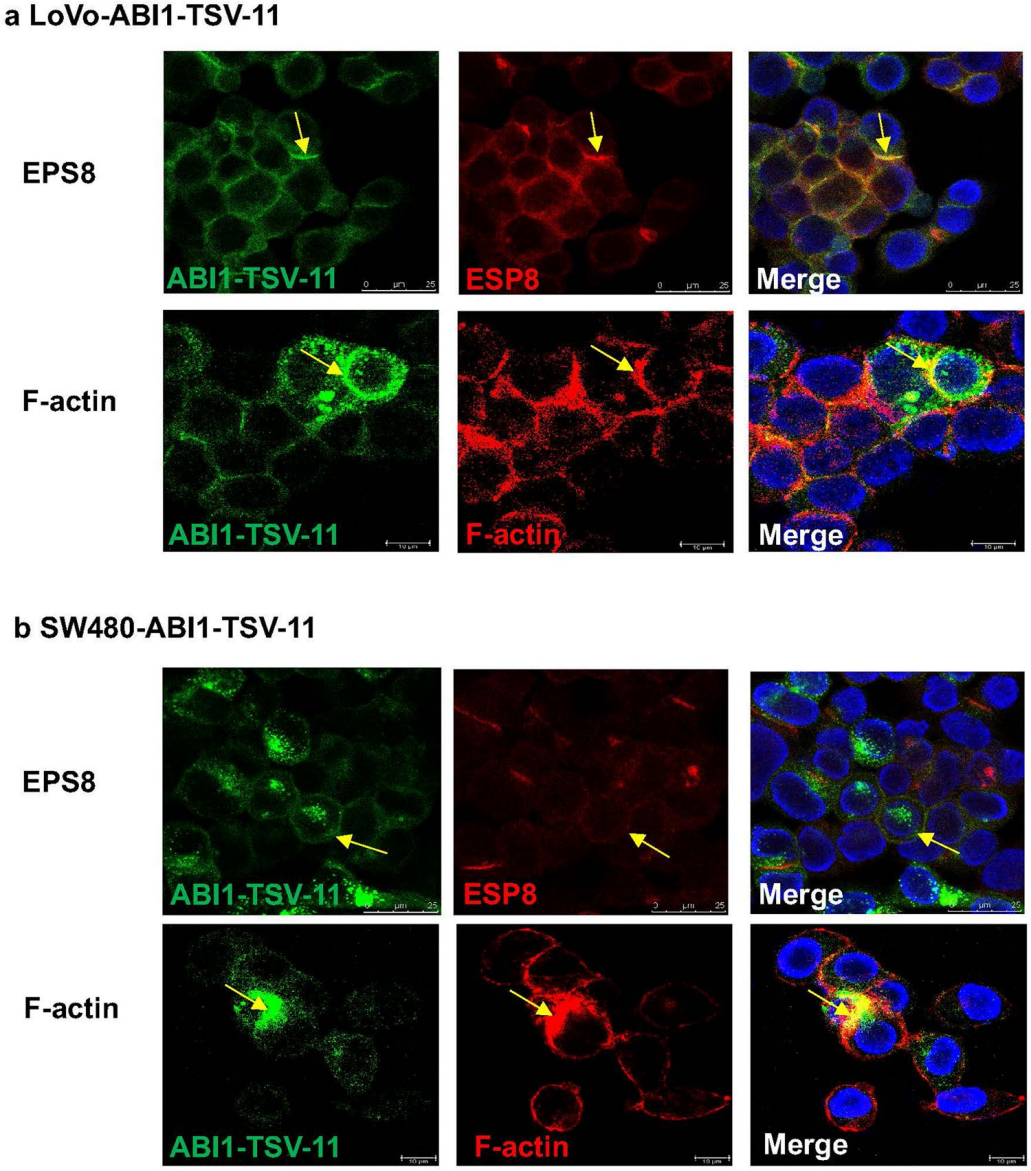
ABI-isoform-11 co-localizes with EPS8 and F-actin in LoVo-ABI1-TSV-11 and SW480-ABI1-TSV-11 cells at the subcellular level. (**a**) The co-localization of ABI1-isoform-11 with EPS8 and F-actin in LoVo-ABI1-TSV-11 cells. (**b**) The co-localization of ABI1-isoform-11 with EPS8 and F-actin in SW480-ABI1-TSV-11 cells (blue: DAPI; green: ABI1-TSV-11; red: EPS8 or F-actin; orange: merge). Yellow arrows indicate co-localization sites.

**Figure 8:**
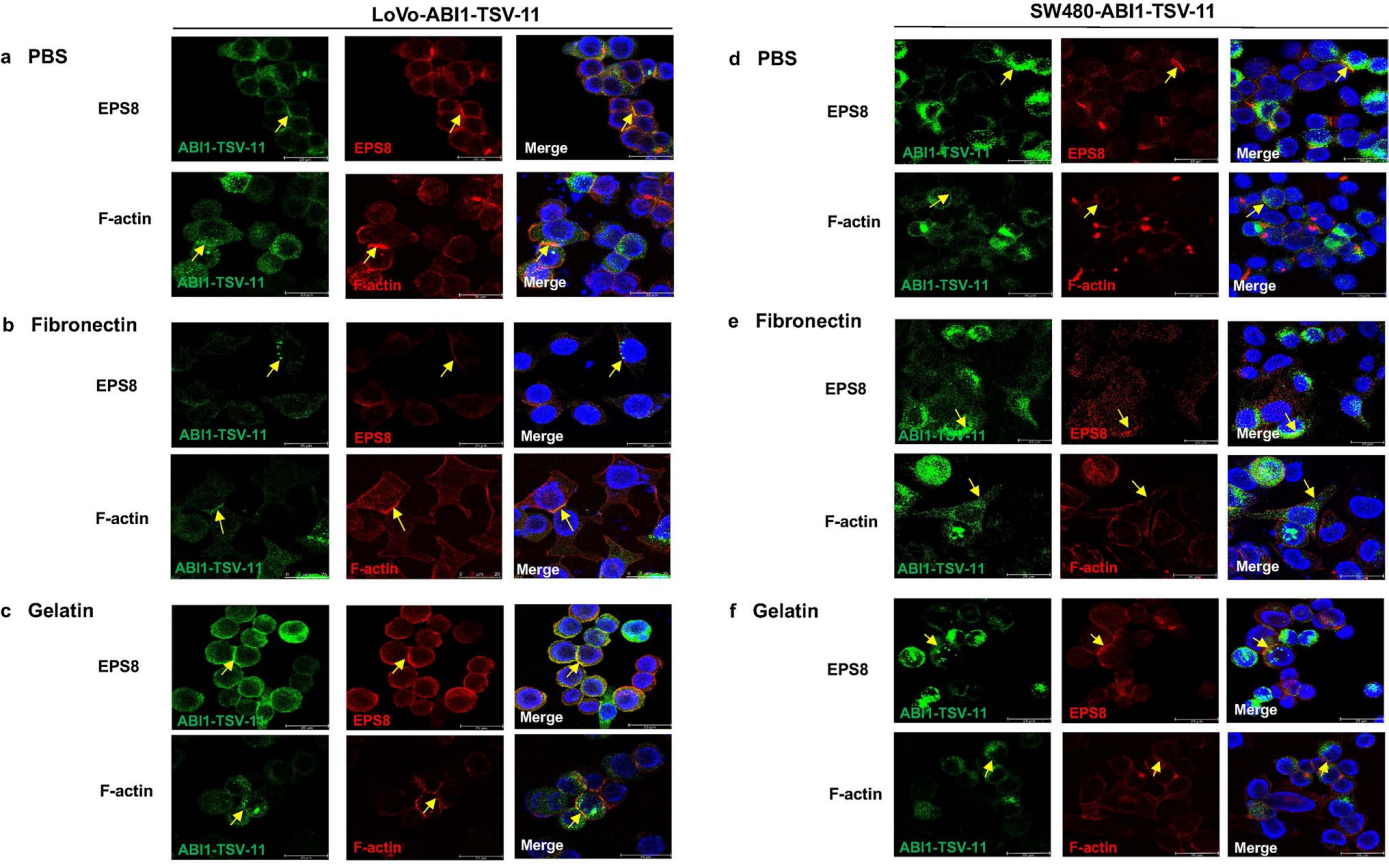
The co-localization of ABI1-isoform-11 with EPS8 and F-actin in LoVo-ABI1-TSV-11 and SW480-ABI1-TSV-11 cells under different coating conditions. (**a**–**c**) The co-localization of ABI1-isoform-11 with EPS8 and F-actin in LoVo-ABI1-TSV-11 cells when coated with PBS, Fibronectin and Gelatin. (**d**–**f**) The co-localization of ABI1-isoform-11 with EPS8 and F-actin in SW480-ABI1-TSV-11 cells when coated with PBS, Fibronectin and Gelatin. (Blue: DAPI; green: ABI1-TSV-11; red: EPS8 or F-actin; orange: merge). Yellow arrows indicate co-localization sites.

ABI1 can regulate actin aggregation and cytoskeleton reconstruction by forming complexes with WAVE2, PI3K, EPS8, and/or N-WASP^17,20,23,24^, and thus play an important role in the metastasis of various malignant tumors including CRC^8–12,27,29,37^. Now we proved that ABI1-isoform-11 interacted only with EPS8 in LoVo and SW480 cells (as shown in Figs. 7, 8), and the co-localization of ABI1-isoform-11 and EPS8 were consistent with the dynamic change of F-actin, specifically with the related trend of lamellar pseudopodium formation and cell extension. Actin dynamics and cytoskeleton changes are the basis of tumor cell adhesion, migration, and invasion^58,59^. The binding of ABI1 and EPS8 could not only affect the localization and/or activity of actin nucleation, regulate the reconstruction of the actin cytoskeleton, and promote the assembly of filopodium structure and actin filaments of human breast cancer cells and mouse melanoma cells^22^, also seal the hook end of EPS8, promote actin capping, and directly induce F-actin-rich structure formation^60^. As shown in Fig. S12, the knockdown of EPS8 in ABI1-TSV-11-expressing LoVo or SW480 cells do affect ABI1-TSV-11-induced protrusions, and the co-localization of ABI1-isoform-11 with F-actin but not cell morphology. Taken these findings together, we believe that ABI1-isform-11 can regulate actin dynamics and cytoskeleton remodeling by interacting with EPS8, thus promoting the adhesion and migration of LoVo and SW480 cells in vitro and accelerating the process of lung metastasis in vivo. However, TCGA database shows that multiple ABI1 TSVs are often expressed in CRC at the same time, and there are potential synergistic or antagonistic effects among different TSVs. Therefore, it is necessary to integrate them with changes in expression, abnormal phosphorylation, and differences in complex formation to systematically elucidate the exact molecular mechanism of ABI1 in CRC metastasis.

In summary, we believe that ABI1-TSV-11 is a specific prognostic factor and therapy target for LsCC, and the current results of this study open up a new field for us to use TSV-specific nucleic acid detection technology^61^ and ASO (antisense oligonucleotide) target technology^62^ for the diagnostic classification, prognostic evaluation, and targeted treatment of CRC metastasis.

## Material and methods

### Acquisition and grouping of clinicopathological information and mRNA sequencing data

We downloaded clinicopathological information of 634 CRC patients from TCGA (version 20,160,128), and ABI1 TSV sequencing data of 382 CRC patients from TSVdb. 339 CRC adenocarcinoma patients with complete survival data were selected into our study, and then divided LsCC (n = 168) and RsCC (n = 154) subgroups according to the primary tumor sites (unknown = 17).

### Cell lines and cell culture

SW480, SW620, LoVo and CRL-1541 cell lines were purchased from the Chinese Academy of Science (Shanghai, China) and ATCC, and cultured in DMEM supplemented with 10% FBS and 1% penicillin & streptomycin.

### Construction of stable and transient cell lines

To establish stable ABI1-TSV-11-overexpressing cell lines, LoVo and SW480 cells were infected with lentivirus at a multiplicity of infection of 30 in the presence of 7 μg/ml polybrene. After puromycin selection for 2 weeks, the expression level of ABI1-TSV-11 was determined by quantitative real-time PCR (qRT-PCR) and western blotting using HA-tag antibody (#3724; CST, MA, USA). To knockdown EPS8 transiently in ABI1-TSV-11-overexpressing cell lines, EPS8 and Non-targeting Control (NC) siRNAs (5’-TCGGTTCTAAAGGATGATATT-3’ and 5’-UUCUCCGAACGUGUCACGUdTdT-3’, respectively) were transfected with lipofectamine 3000. After 48 h, the expression level of EPS8 was determined by qRT-PCR.

### qRT-PCR

qRT-PCR was performed as previously described^9^. The primers were listed in Supplement Materials (Table S1) and the amplification was conducted and analyzed by CFX96 (Bio-Rad, Hercules, CA, USA).

### Integrins mRNA expression profile of LoVo and SW480 cells

RNA sequencing was performed using the Illumina HiSeq 2000 RNA Sequencing platform ((MGI, Shenzhen, P. R. China)) and the integrins mRNA expression profile of LoVo and SW480 cells was constructed with normalized FPKM count.

### Proliferation, adhesion, migration, and invasion analyses of cells in vitro

Cell proliferation assays were performed using Cell Counting Kit-8 (CK04-100, CCK8; Dojindo, Rockville, MD, USA). Adhesion assays were conducted with 24-well plates (3524; Corning) coated with different materials, namely, PBS, (21–040-CV; Corning), 0.2% BSA (A-1933; Sigma), fibronectin (5 μg/ml) (F1141; Sigma), collagen I (C9879; Sigma), and 0.1% gelatin (ES-006-B; Millipore, MA, USA). Migration and invasion assays were performed with a 24-well Transwell chamber with 8.0-μm-pore polycarbonate filter inserts (3422; Corning). We seeded cells on the upper chambers without or with Matrigel (356,234; Corning, 300 μg/ml). We counted the cells that had migrated to the lower side of the insert membrane at 4 h (LoVo-ABI1-TSV-11 and parental cells; LoVo-ABI1-TSV-11-EPS-siRNA and LoVo-ABI1-TSV-11-NC-siRNA) or 8 h (SW480-ABI1-TSV-11 and parental cells; SW480-ABI1-TSV-11-EPS-siRNA and SW480-ABI1-TSV-11-NC-siRNA) after seeding cells, and counted the cells that had invaded to the lower side of the insert membrane at 8 h (LoVo-ABI1-TSV-11 and parental cells; LoVo-ABI1-TSV-11-EPS-siRNA and LoVo-ABI1-TSV-11-NC-siRNA) and 14 h (LoVo-ABI1-TSV-11 and parental cells;SW480-ABI1-TSV-11-EPS-siRNA and SW480-ABI1-TSV-11-NC-siRNA) after seeding.

### Establishment of colorectal cancer lung metastasis model

All animal care and handling procedures were were approved by the Ethics Committee of Peking University People’s Hospital BALB/c nude mice (6–8 weeks old; Charles River, Beijing, China) were kept in SPF conditions and randomly assigned into four groups (six mice per group). LoVo-ABI1-TSV-11, LoVo-EV, SW480-ABI1-TSV-11, and SW480-EV (each 1 × 10^6^) cells were injected into the tail veins of nude mice. At 16 weeks after injection, all tested mice were sacrificed with the signs of cachexia appearing, and qualitative and quantitative analyses of lung metastases were conducted by hematoxylin and eosin (H&E) staining, Nano-Zoomer Digital Pathology S360 (Hamamatsu Photonics, Shizuoka, Japan), and ImageJ (National Institutes of Health, USA).

### Western blotting and co-immunoprecipitation

Western blot and immunoprecipitation analysis were performed as previously described^9^. The antibodies were listed in Supplement Materials (Table S2).

### Immunofluorescence

LoVo-ABI1-TSV-11 and SW480-ABI1-TSV-11, LoVo-ABI1-TSV-11-EPS-siRNA and LoVo-ABI1-TSV-11-NC-siRNA, SW480-ABI1-TSV-11-EPS-siRNA and SW480-ABI1-TSV-11-NC-siRNA cell lines were cultured on chambers (154,526; Thermo Fisher Scientific, MA, USA). The cells were fixed in 4% paraformaldehyde in PBS for 10 min and permeabilized with 0.2% Triton X-100 in PBS for 5 min. Nonspecific binding was blocked by incubation with normal goat serum. Then cells were incubated with the primary antibody EPS8 or Phalloidin-iFluor-647 working solution. After washing with PBS, cells were incubated with TRITC-Labeled Goat Anti-Rabbit IgG (skip this step when staining F-actin), followed by counterstaining with DAPI. The images were acquired by confocal laser scanning microscopy (CLSM, TCS-SP8/STED 3X; Leica, Germany). The information of antibodies and dyes were listed in Supplement Materials (Table S2).

### Statistical analysis

Statistical analysis and graph drawing were performed using SPSS 20.0 (IBM, Chicago, IL, USA) and GraphPad Prism 8.0 (La Jolla, CA, USA). X-tile 3.6.1 (Yale University School of Medicine, New Haven, CT, USA) was used to confirm the most suitable cut-off points to define low and high ABI1-TSV-11 expression. Chi-square test was used to compared two groups, and one-way ANOVA was used to compare three or more groups. Kaplan–Meier analysis and log-rank test were used to survival analysis. The Cox proportional hazards regression model was used to univariate and multivariate analysis to determine the effect of ABI1-TSV-11 on OS. *P*-values < 0.05 were considered to be statistic significant. All *p*-values correspond to two-sided significance tests.

### Approval for animal experiments

The animal experiments were approved by the Ethics Committee of Peking University People’s Hospital and all animal care and handling procedures were performed according to the National Institutes of Health's Guide for the Care and Use of Laboratory Animals. The study was carried out in compliance with the ARRIVE guidelines 2.0 (https://arriveguidelines.org).

## Supplementary Information

Supplementary Figures and Tables.
