# Supplementary material for: The roles and prognostic significance of ABI1-TSV-11 expression in patients with left-sided colorectal cancer
Source: Sci Rep. 2021 May 24;11:10734. doi: 10.1038/s41598-021-90220-8 (PMC8144562; doi:10.1038/s41598-021-90220-8)
Supplement: Supplementary file 1 — Supplementary Figures and Tables. [file 41598_2021_90220_MOESM1_ESM.pdf]

The Roles and Prognostic Significance of ABI1-TSV-11 Expression in Patients with Left-sided Colorectal Cancer

Yu Zhang[1,2,+], Zhaohui Zhong[3,+], Mei Li[1], Jingyi Chen[1,2], Tingru Lin[1,2], Jie Sun[1], Di Wang[1], Qing Mu[1], Huiting Su[1], Na Wu[1], Aiyu Liu[1], Yimeng Yu[1], Menglei Zhang[4],Yulan Liu[2], Jingzhu Guo[5,*], Weidong Yu[1,*]

·Authors' affiliations:

[1]Department of Central Laboratory & Institute of Clinical Molecular Biology, Peking University People's Hospital, Beijing, China

[2]Department of Gastroenterology, Peking University People's Hospital, Beijing, China

[3]Department of General Surgery, Peking University People's Hospital, Beijing, China

[4]Department of Animal Laboratory, Peking University People's Hospital, Beijing, China

[5]Department of Pediatric, Peking University People's Hospital, Beijing, China

***Corresponding authors:**

Jingzhu Guo, guojingzhu@pkuph.edu.cn

Weidong Yu, weidongyu@bjmu.edu.cn

+Yu Zhang and Zhaohui Zhong contributed equally to this study

Supplementary material

Fig.S1

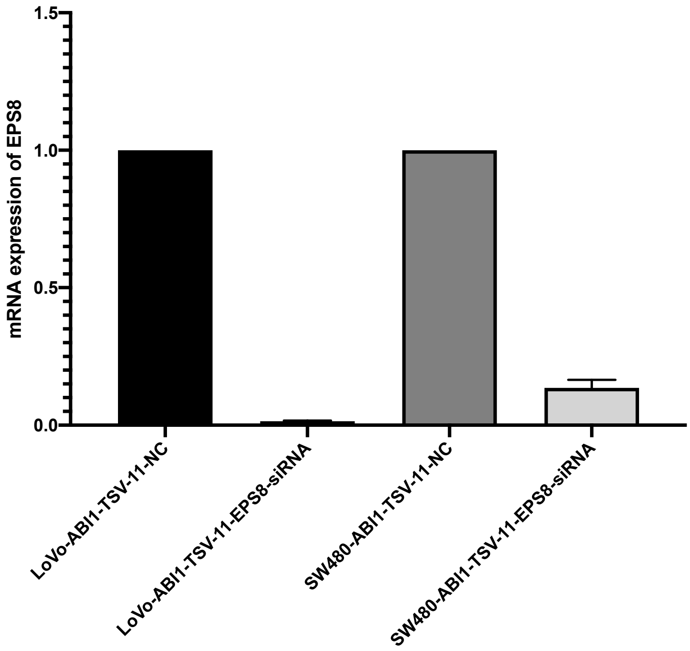

Fig. S1. Establishment of EPS8-knockdown cell lines by EPS8-siRNA in ABI1-TSV-11 overexpressing LoVo and SW480 cell lines.

qRT-PCR analysis using *EPS8* ***specific*** primers of in LoVo-ABI1-TSV-11 and SW480-ABI1-TSV-11 cell lines after EPS8 and NC siRNAs transfection.

Fig.S2

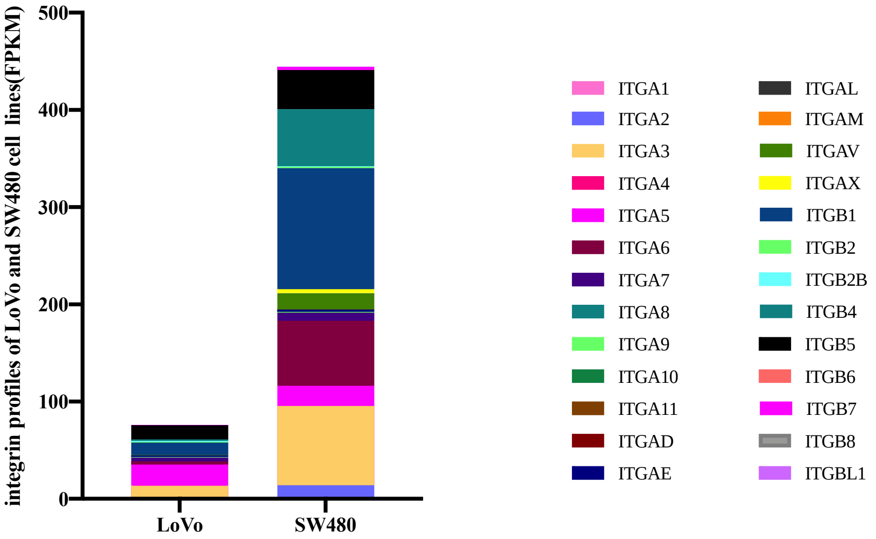

Fig. S2. integrins mRNA expression profiles of LoVo and SW480 cell lines using RNA sequencing data.

Fig. S3

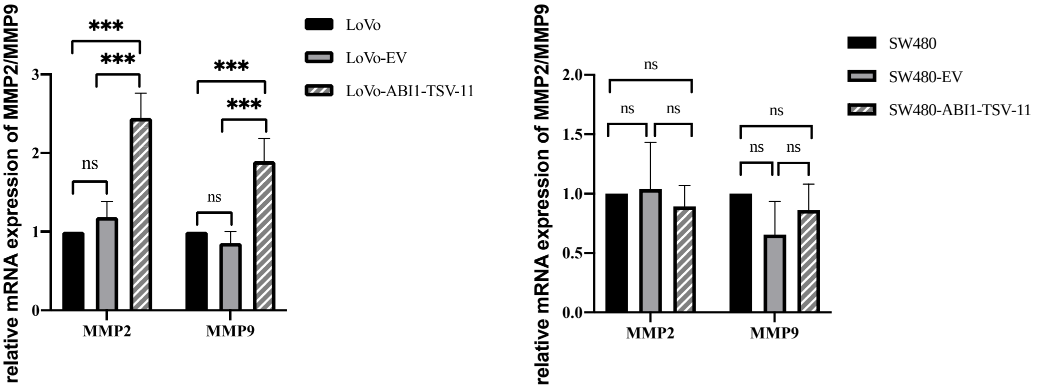

Fig. S3. mRNA expression of MMP2 and MMP9 of LoVo-ABI1-TSV-11, SW480-ABI1-TSV-11 and their corresponding controls

Fig.S4

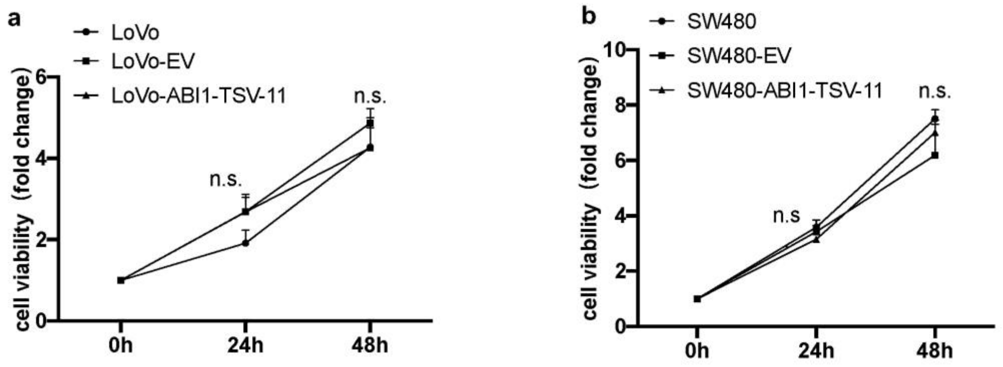

Fig. S4. **ABI1-TSV-11 overexpression has no effects on proliferation and cell cycle in LoVo and SW480 cell lines *in vitro*.**

a, Proliferation analysis of LoVo-ABI1-TSV-11 cell line and controls using Cell Counting Kit-8. b, Proliferation analysis of SW480-ABI1-TSV-11 cell line and controls using Cell Counting Kit-8. Data are presented as the mean ± S.D. of three or four experiments (one-way ANOVA, *P < 0.05, **P < 0.01, ***P < 0.001, *ns*, not significant).

Fig. S5

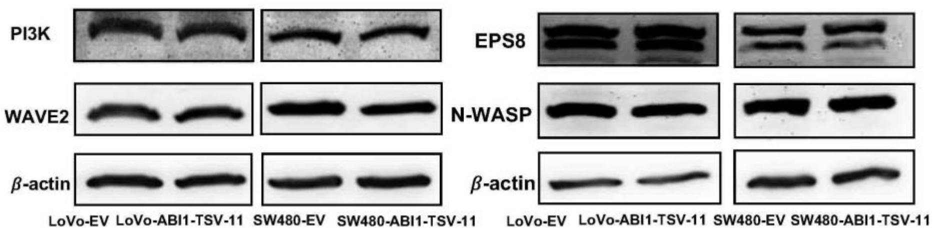

a

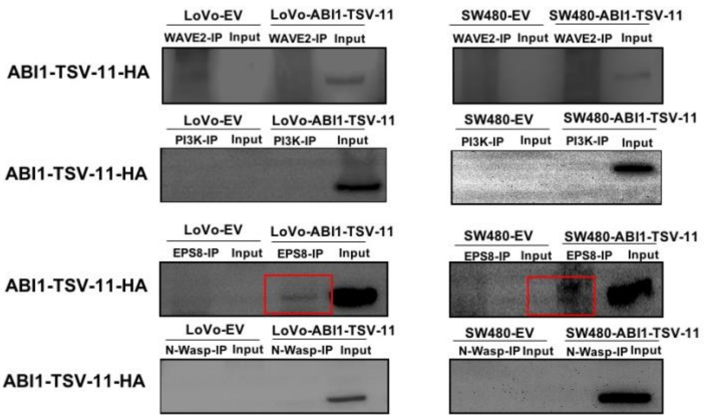

b

Fig.S5. WAVE2, PI3K, EPS8, and N-WASP protein expression in LoVo-ABI1-TSV-11, LoVo-EV, SW480-ABI1-TSV-11, SW480-EV cells, and the interaction of ABI1-isoform-11 with EPS8.

a, Protein expression analysis of WAVE2, PI3K, EPS8, and N-WASP in LoVo-ABI1-TSV-11, SW480-ABI1-TSV-11, and their corresponding control cells. b, ABI1-isoform-11 interacts with ESP8（shown in red boxes）, not WAVE2, PI3K and N-WASP in LoVo-ABI1-TSV-11 and SW480-ABI1-TSV-11 cells.

Fig.S6

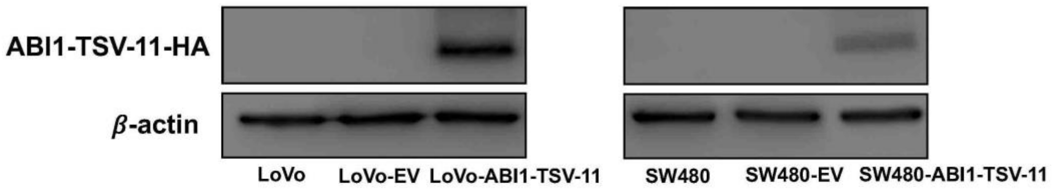

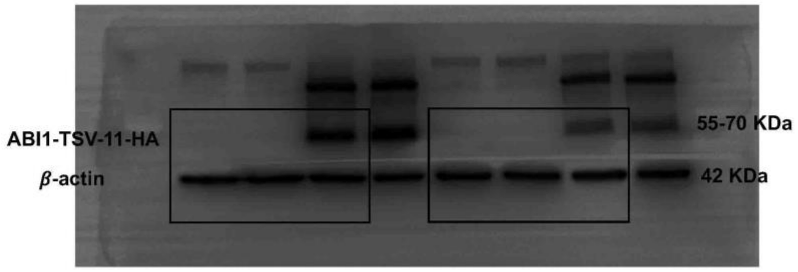

Fig.S6. Full length western blot of Fig.2d

Fig.S7

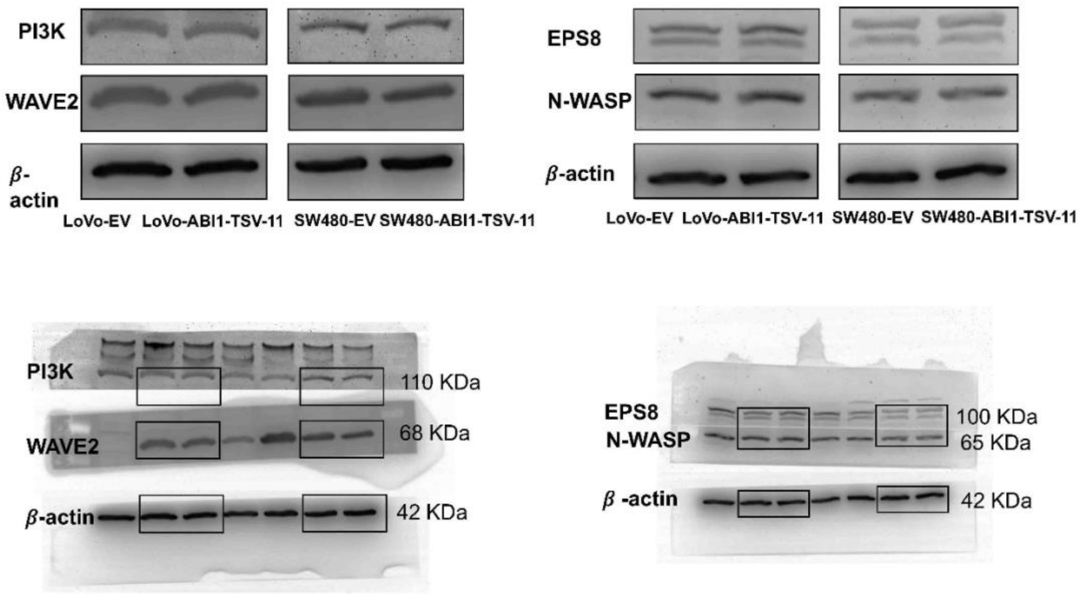

Fig.S7. Full length western blot of Fig.S5a

Fig.S7

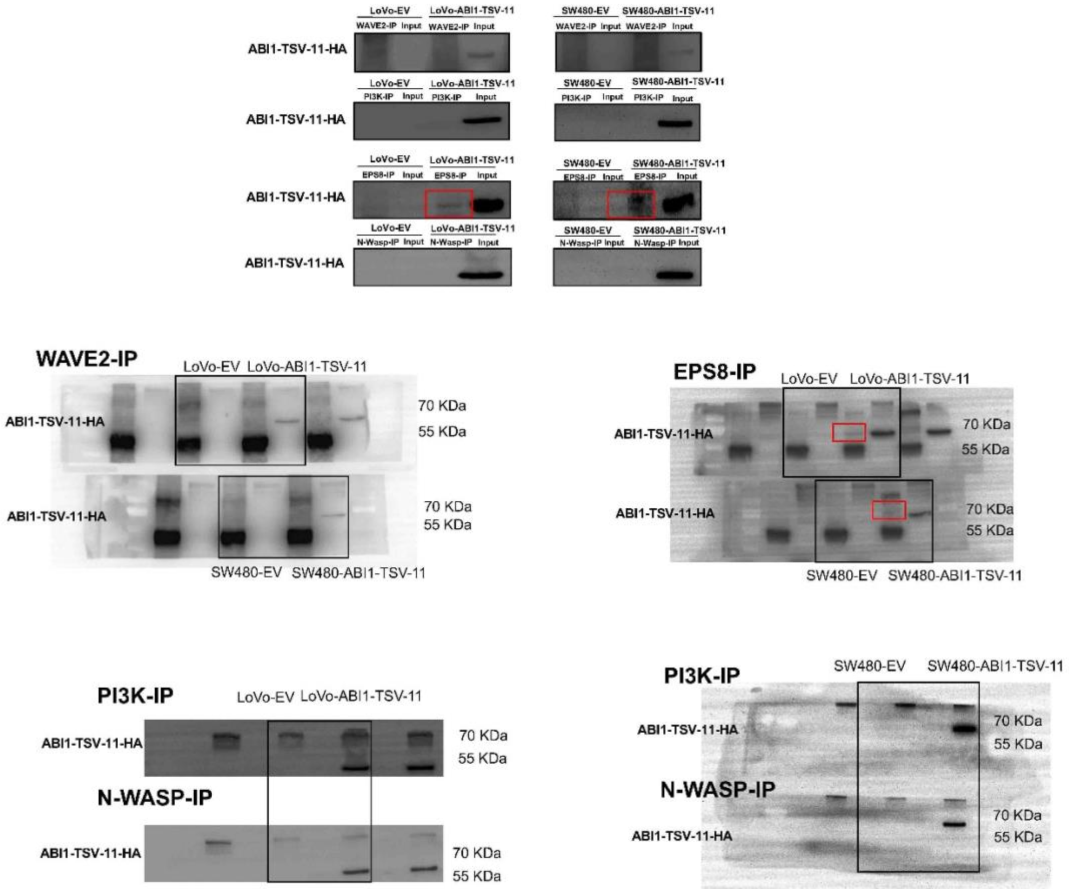

Fig.S8. Full length co-immunoprecipitation of Fig.S5b

Fig. S9

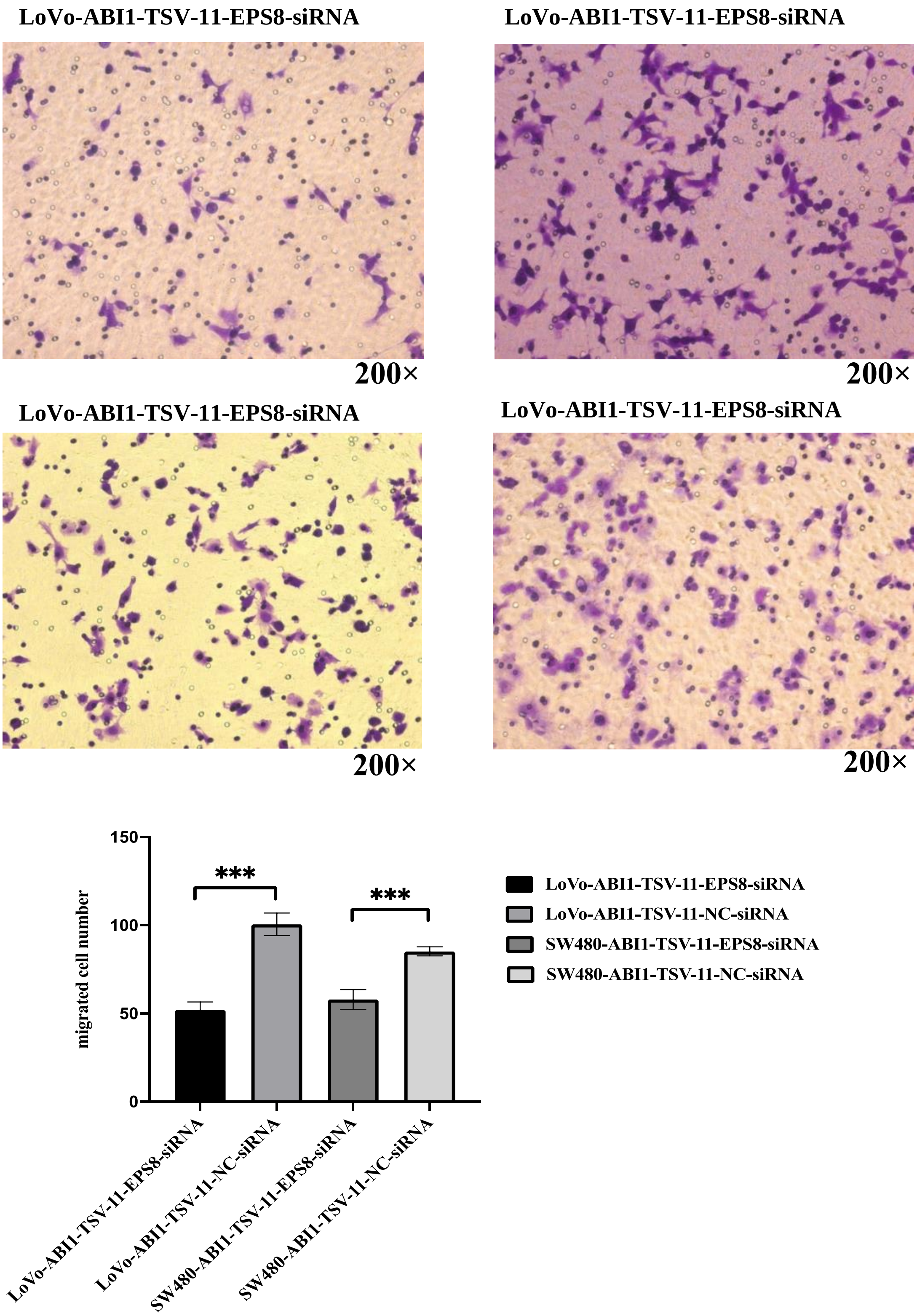

Fig.S9 EPS8 kncokdown attenuates migration of LoVo-ABI1-TSV-11 and SW480-ABI1-TSV-11 cells in vitro (*P < 0.05, **P < 0.01, ***P < 0.001, ns, not significant)

Fig. S10

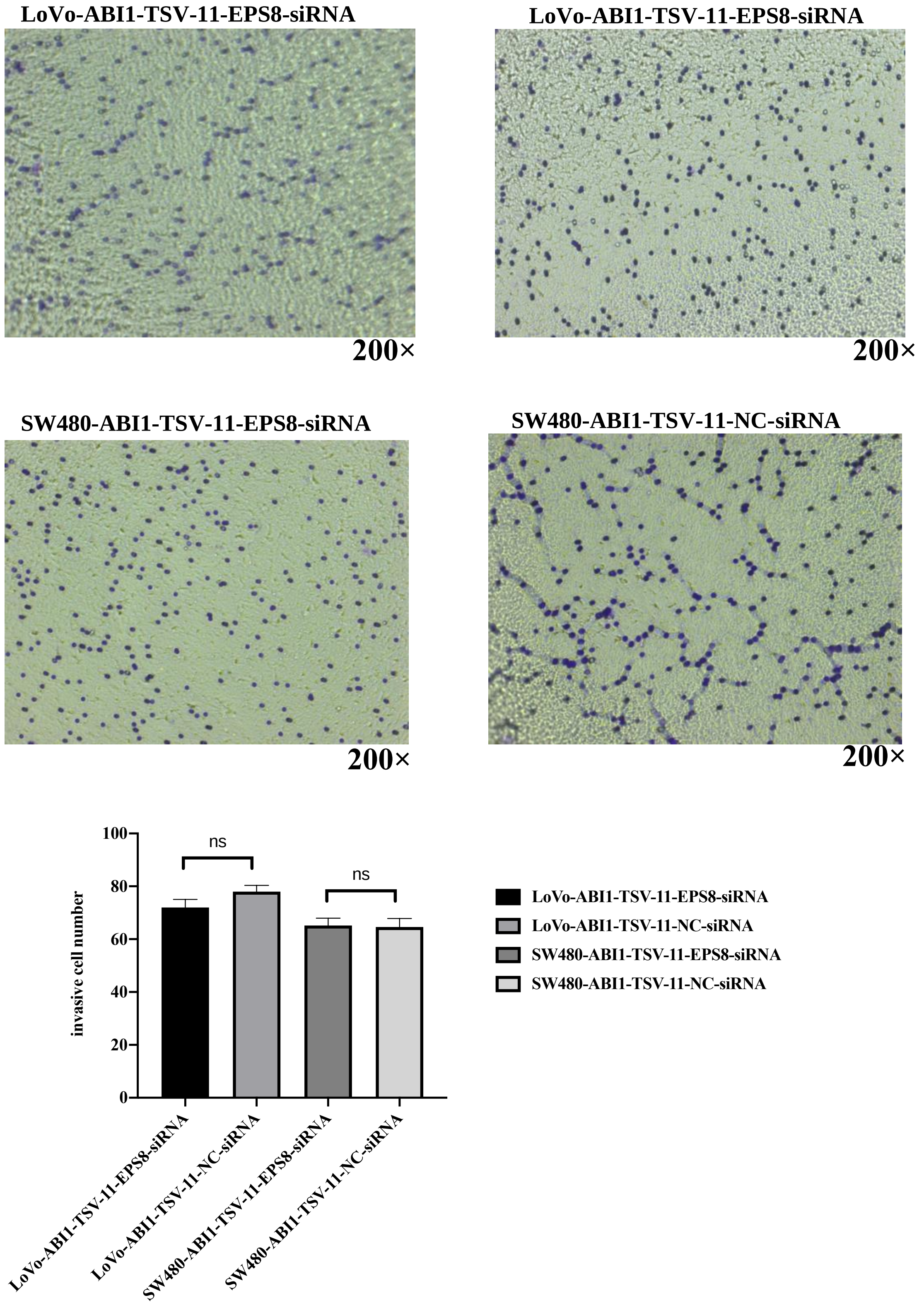

Fig. S10 EPS8 Kncokdown does not change invasion of LoVo-ABI1-TSV-11 and SW480-ABI1-TSV-11 cell lines in vitro. (*P < 0.05, **P < 0.01, ***P < 0.001, ns, not significant)

Fig. S11

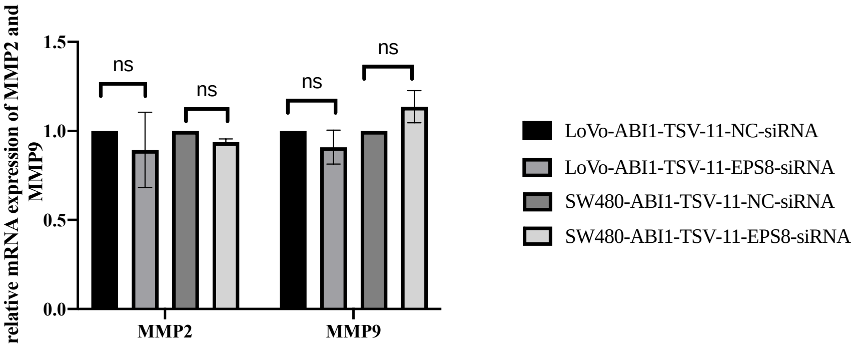

Fig. S11 mRNA expression of MMP2 and MMP9 of LoVo-ABI1-TSV-11-EPS8-siRNA, SW480-ABI1-TSV-11-EPS8-siRNA and their corresponding controls (*P < 0.05, **P < 0.01, ***P < 0.001, ns, not significant)

Fig. S12

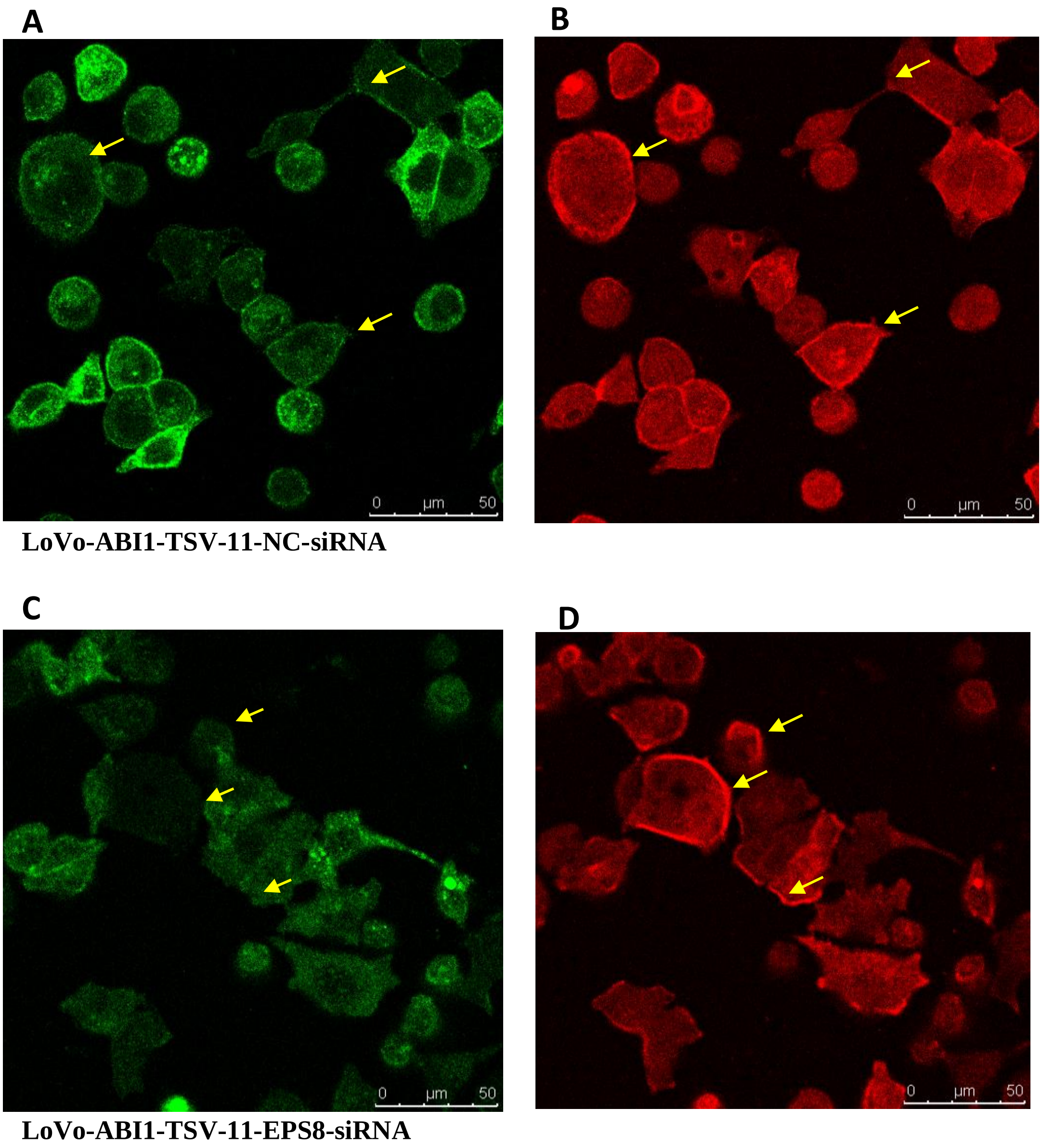

E F

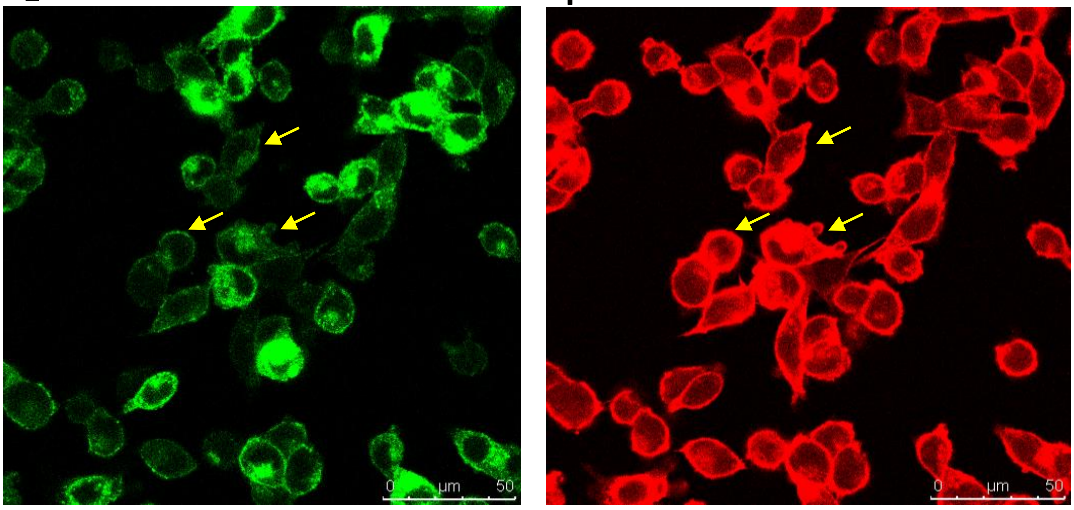

SW480-ABI1-TSV-11-NC-siRNA

G H

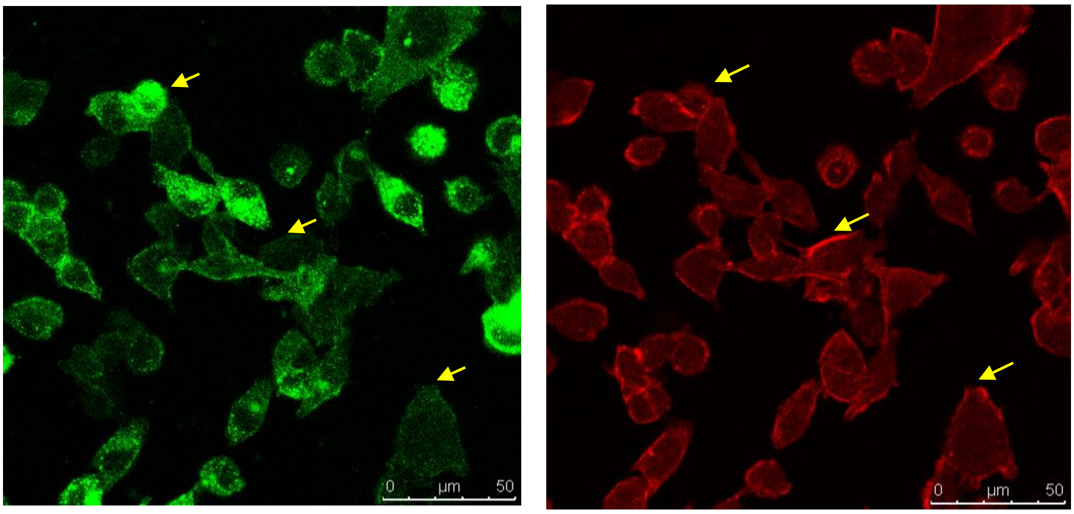

Fig.S12 knockdown EPS8 attenuates the colocalization of ABI-isoform-11（Green）and F-actin （Red）in LoVo-ABI1-TSV-11 cell lines（A-D） and SW480-ABI1-TSV-11 cell lines（E-H）at the subcellular level.

Table S1 The primers for qRT-PCR assay.

Primer Name	Primer Sequence (5'-3')
β-actin Forward primer	5'- CATGTACGTTGCTATCCAGGC-3'
β-actin Reverse primer	5'-CTCCTTAATGTCACGCACGAT-3'
ABI1 Forward primer	5'-ACACTGGGACGGAATACTCCTTAT-3'
ABI1 Reverse primer	5'-ACACTGGGACGGAATACTCCTTAT-3'
MMP2 Forward primer	5'-AGCGAGTGGATGCCGCCTTTAA-3'
MMP2 Reverse primer	5'-CATTCCAGGCATCTGCGATGAG-3'
MMP9 Forward primer	5'-GCCACTACTGTGCCTTTGAGTC-3'
MMP9 Reverse primer	5'-CCCTCAGAGAATCGCCAGTACT-3'
EPS8 Forward primer	5'-GCCAACTTCTAATCGCCATA-3'
EPS8 Reverse primer	5'-TCACTGTTGTTCCTTGCTAC-3'

Table S2 Antibodies or dyes used in this study.

Name	Article number	Corporation name	Country	Dilution ratio
β-actin	TA-09	Zhongshan Golden-bridge Biotechnology	China	1:1000（WB）
ABI1	sc-30038	Santa Cruz Biotechnology	USA	1:200(WB)
HA-Tag	#3724	Cell Signaling Technology	USA	1:1000(WB)
WAVE2	07-410	Millipore	USA	1:1000 (WB)<br>1:50（IP）
PI3K	#4228	Cell Signaling Technology	USA	1:1000 (WB)<br>1:50（IP）
ESP8	#43114	Cell Signaling Technology	USA	1:1000 (WB)<br>1:50（IP）<br>1:100（IF）
N-WASP	#4848	Cell Signaling Technology	USA	1:1000 (WB)<br>1:50（IP）
TRIRC-labeled Goat Anti-Rabbit IgG	ZF-0316	Zhongshan Golden-bridge Biotechnology	China	1:50（IF）
Phalloidin-iFluor-647	ab176759	Abcam	UK	1X（IF）
DAPI	F01001-00	GP Medical Technologies	China	10ug/ml（IF）

BioMed Proofreading® LLC *(Since 2003)*

You write it, we polish it

info@biomedproofreading.com
www.biomedproofreading.com
WeChat: copyeditor

May 18, 2020

To Whom It May Concern:

This letter confirms that the manuscript entitled *Elevated ABI1-TSV-11 expression is related to lymph node metastasis and poor prognosis of left-sided colorectal cancer, and promotes LoVo and SW480 cancer cell migration in vitro and lung metastasis in vivo by interacting with EPS8* by Dr. Yu et al has been copyedited by native English speakers with a related biomedical background in ***BioMed Proofreading® LLC***.

Sincerely,

BioMed Proofreading®, LLC

www.biomedproofreading.com

info@biomedproofreading.com

BIOMED PROOFREADING, LLC LIMITED LIABILITY COMPANY SEAL STATE OF OHIO

BioMed Proofreading® is a leading English-language copy-editing company founded in 2003 in the U.S.A. Our focus is proofreading and editing of biomedical manuscripts, proposals and grant applications. We comprised medical and life science professionals, with extensive experience as authors and referees for peer-reviewed biomedical journals. During the past eight years, we have provided services for scientists throughout the world, and we are proud to have earned a stellar reputation. As an English editing company we have been evaluated and recommended by ***Brazilian Society of Zoology***, ***Journal of Human Kinetics*** (Poland), ***Kyushu University*** (九州大学, Japan), ***Phycology Research Group*** (Czech Republic), ***Chinese Medical Journal***, ***Brazilian Journal of Microbiology***, ***Journal of Biomedical Nanotechnology*** (American Scientific Publishers), ***Science of Advanced Materials*** (American Scientific Publishers), ***Brazilian Journal of Medical and Biological Research***, ***Acute and Critical Care*** (South Korea), ***Korean Journal of Critical Care Medicine***, and ***Shengjing Hospital of China Medical University***（中国医科大学附属盛京医院）.

Crown Centre, Suite 60093, 5005 Rockside Road, Cleveland, Ohio 44131, USA.
Tel: (440)584-0186; Fax: (216)373-5499.

Fig.S13. Certificate for English copyediting service.
